# Osmotic Remodeling of Extracellular Vesicles for Precision Nanomedicine

**DOI:** 10.1002/smll.202510793

**Published:** 2026-07-01

**Authors:** Francesca Susa, Lorenzo Arnaboldi, Veronica De Giorgis, Marco Ghirimoldi, Elettra Barberis, Marco Brucale, Francesco Valle, Mario Malerba, Marta Vallino, Angelo Musicò, Roberto Frigerio, Alessandro Gori, Vera Mugoni, Claudia Voena, Roberto Pisano, Fabrizio Pirri, Silvia Arpicco, Marcello Manfredi, Tania Limongi

**Affiliations:** ^1^ Department of Drug Science and Technology University of Torino Torino Italy; ^2^ Department of Pharmacological and Biomolecular Sciences “Rodolfo Paoletti” Università degli Studi di Milano Milano Italy; ^3^ Department of Translational Medicine Center For Allergic and Autoimmune Diseases University of Piemonte Orientale Novara Italy; ^4^ Department of Sciences and Technological Innovation Center For Allergic and Autoimmune Diseases University of Piemonte Orientale Alessandria Italy; ^5^ Consiglio Nazionale delle Ricerche—Istituto per lo Studio dei Materiali Nanostrutturati (CNR‐ISMN) Bologna Italy; ^6^ Istituto Nazionale di Ricerca Metrologica (INRIM) Torino Italy; ^7^ Consiglio Nazionale delle Ricerche (CNR) di Torino Torino Italy; ^8^ Istituto Di Scienze e Tecnologie Chimiche “Giulio Natta” National Research Council of Italy (SCITEC‐CNR) Milano Italy; ^9^ Department of Molecular Biotechnology and Health Sciences Molecular Biotechnology Center University of Torino Torino Italy; ^10^ Department of Applied Science and Technology Politecnico Di Torino Torino Italy; ^11^ Institute for Molecular and Translational Cardiology (IMTC) IRCCS Policlinico San Donato Milano Italy

**Keywords:** drug delivery, extracellular vesicles, osmotic lysis, personalized medicine

## Abstract

Extracellular vesicles (EVs) are increasingly explored as diagnostic and therapeutic platforms. Translation is constrained by protocols that must balance efficacy and safety, especially for EVs from tumor or immortalized cells that may carry oncogenic or immunostimulatory cargo. This study presents a rapid and reproducible engineering of EVs using bidistilled water as the sole reagent. The hypotonic environment induces osmotic lysis of EVs isolated from B lymphocytes, generating a remodeled vesicle population. The process markedly reduces a broad spectrum of intraluminal components with potential immunogenic or tumor‐promoting activity, while preserving the vesicular membrane scaffold. Osmotic remodeling is associated with reorganization of membrane‐associated lipids and proteins and with a preferential uptake of the remodeled vesicles by tumor cells over non‐malignant counterparts in our in vitro model. The engineered EVs retain structural integrity and are amenable, at a proof‐of‐concept level, to reloading with defined bioactive compounds, suggesting their potential as customizable delivery systems. Overall, this osmotic remodeling strategy provides a versatile technological platform for the generation of membrane‐derived vesicles with tunable properties and promising applicability in drug delivery, nanodiagnostics, and immune modulation, whose translational performance and safety will need to be established in source‐ and indication‐specific preclinical studies.

## Graphical abstract

Hypotonic lysis in bidistilled water is used to remodel B lymphocyte–derived extracellular vesicles into cargo‐depleted, structurally preserved nanovesicles. The process reduces intraluminal proteins and nucleic acids, reshapes membrane composition, and enhances tumor‐cell uptake in vitro, while allowing proof‐of‐concept reloading with exogenous agents, outlining a scalable platform for future EV‐based nanomedicine applications.

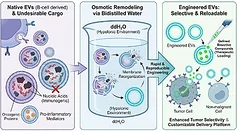

## 1 Introduction

Extracellular vesicles (EVs) are a heterogeneous population of lipid bilayer‐enclosed nanoparticles secreted by virtually all eukaryotic and prokaryotic cells. Since their discovery in the 1980s, EVs have been increasingly recognized for their central role in intercellular communication, contributing to a wide range of physiological and pathological processes. Their ability to transfer lipids, proteins, nucleic acids, and metabolites to recipient cells has revolutionized the fields of diagnostics and nanomedicine, enabling early disease detection and the development of innovative therapeutic strategies [1].

In recent years, the scientific community has focused intensively on the potential of EVs as both diagnostic biomarkers and therapeutic agents [2, 3, 4]. This interest is reflected in the dramatic increase in EV‐related publications, now approaching 50,000, and in the activation of approximately 300 clinical trials investigating their application in human diseases [5, 6, 7, 8]. These studies span several domains, from oncology and immunology to regenerative medicine and neurosciences, and their applications in nanotechnology are significantly increasing [9, 10, 11, 12, 13].

EVs mirror the molecular signature of their parental cells, including membrane‐associated proteins and lipids, as well as internal cargo such as nucleic acids, some species lipids, and cytosolic factors [14, 15]. Functionally, they regulate homeostasis and immune responses and play critical roles in disease progression, including cancer metastasis, cardiovascular dysfunction, and neurodegeneration [2, 16, 17]. Their ability to bind to specific recipient cells through surface molecules such as tetraspanins, integrins, and proteoglycans allows them to promote targeted delivery and cellular uptake [2, 18, 19].

In addition to their diagnostic relevance, EVs have emerged as promising therapeutic tools. Their inherent biocompatibility, low immunogenicity, high stability, and ability to cross biological barriers, including the blood–brain barrier, make them attractive vehicles for drug delivery [20, 21, 22, 23, 24, 25, 26, 27].

EVs derived from biological fluids or cultured cells such as mesenchymal stromal cells (MSCs), dendritic cells, macrophages, and hematopoietic stem cells have demonstrated therapeutic effects in various disease models, including cancer, neurological, inflammatory, and autoimmune disorders [22, 28, 29, 30, 31, 32, 33]. In preclinical models of ischemic stroke, systemic administration of MSC‐derived EVs has been shown to promote functional recovery and neurovascular plasticity after brain injury, while EVs derived from adipose tissue MSCs also improve neurological outcomes when administered in the subacute phase of stroke [34, 35]. In the cardiovascular field, EVs from MSCs and other healthy tissues attenuate cardiac dysfunction, reduce inflammation and fibrosis, and enhance angiogenesis in murine models of myocardial infarction [36]. In oncology, EVs have been exploited as nanocarriers for chemotherapeutic drugs, enabling targeted delivery and reduced systemic toxicity in tumor models [37]. Moreover, MSC‐derived EVs have been reported to alleviate graft‐versus‐host disease while preserving graft‐versus‐leukemia activity in preclinical and early clinical settings, underscoring their translational potential in immune‐mediated disorders [38]. Collectively, these studies provide direct experimental evidence that EVs can exert therapeutic functions in vivo and can be engineered as biologically active nanocarriers for precision interventions. Despite these encouraging findings, the direct use of native EVs as therapeutic products still faces important limitations, including the presence of oncogenic or immunomodulatory cargo, batch‐to‐batch variability, and the co‐isolation of soluble contaminants and protein aggregates, which raise concerns regarding safety, standardization, and regulatory compliance.

Cancer‐derived EVs have demonstrated long‐range organotropism, strong immunomodulatory activity, and efficient tumor targeting, all of which make them attractive for therapeutic delivery [39, 40]. However, their endogenous cargo often contains oncogenic or immunosuppressive molecules, which raises safety concerns and limits their clinical application [41, 42]. To address these limitations, a variety of engineering strategies have been employed to load EVs with therapeutic cargo or modify their surface properties. These include post‐isolation techniques such as passive co‐incubation and active methods like electroporation, sonication, and freeze–thaw cycles, as well as pre‐isolation approaches involving the genetic modification of donor cells [13, 43, 44]. Surface engineering can improve targeting capabilities and pharmacokinetics, for instance, through the addition of ligands or anti‐fouling polymers such as polyethylene glycol (PEG), or by preventing clearance via phagocytosis [30, 45, 46, 47, 48]. A further strategy involves the creation of hybrid vesicles through the fusion of EVs membranes with synthetic liposomes, thereby enhancing both stability and functional versatility [49, 50, 51, 52]. Despite such innovations, key challenges remain that hinder the broader clinical use of EVs. These include difficulties in scaling up production from stem or primary cells, achieving sufficient yield and purity, and ensuring consistency in cargo content. Some methods aim to boost yield by altering environmental conditions, such as pH, shear stress, temperature, oxygen tension, or ethanol treatment, but these must be applied with care as they may also impact EVs integrity and function [30, 53, 54]. Importantly, managing the co‐existence of endogenous and exogenous cargo within engineered EVs is a critical issue, as unwanted interactions may result in reduced therapeutic specificity, off‐target effects, or potential immunogenicity [20, 40].

Building on our previous experience with more disruptive EV engineering strategies, including repeated freeze–thaw cycles [13], which proved effective as proof‐of‐concept tools but were associated with limited vesicle recovery, labor‐intensive workflows, and a measurable impact on EV integrity, we sought to develop a gentler, purely water‐based, and more scalable remodeling strategy. In the following study, we describe an innovative, fast, reproducible, and sustainable method for EVs engineering that aims to address several of these limitations and may facilitate future translation toward clinical applications. The strategy is based on osmotic lysis, which involves the rapid dilution of EVs in a hypotonic aqueous solution, such as double‐distilled water, to induce membrane disruption. The mechanism relies on the ionic imbalance between the inside and outside of the vesicle, leading to a net water influx, swelling, and eventual rupture of the lipid bilayer. While cells can adapt to gradual osmotic changes through ion transport mechanisms, a sudden hypotonic shock, as used in this protocol, causes irreversible membrane lysis [55, 56]. This approach, previously established for erythrocyte ghosts and synthetic lipid vesicles [57, 58, 59, 60, 61, 62], has been adapted and optimized here for EVs isolated via ultracentrifugation. However, because it relies on a fundamental osmotic gradient across the vesicle membrane, the same conceptual strategy may, in principle, be adapted to EVs obtained using other commonly employed isolation methods and biological sources, provided that the basic treatment conditions are appropriately optimized for each specific source. Here, we demonstrate that subjecting EVs to a controlled hypo‐osmotic shock induces membrane disruption followed by spontaneous reassembly into structurally stable nanovesicles. This fast and reproducible method results in a vesicle population with a higher particle concentration and improved purity. Notably, it allows for the substantial depletion of intraluminal cargo such as proteins and nucleic acids, components often associated with tumorigenic or immunogenic risks, thus generating membrane‐derived vesicles largely depleted of unwanted internal content. This process opens new possibilities for tunable drug delivery applications with potentially improved safety profiles, particularly when exploiting the natural targeting properties of tumor‐derived EVs membranes. Furthermore, the lysis and reassembly protocol is compatible, at a proof‐of‐concept level, with the concurrent loading of exogenous cargo, expanding the potential for therapeutic customization. Remarkably, the structural and compositional lipid and protein rearrangements of the vesicle membranes resulting from lysis lead to enhanced cellular uptake, especially in tumor cells, suggesting that the engineered vesicles may offer improved targeting specificity. Together, these results support the potential use of osmotic lysis as a versatile strategy to obtain functional, customizable, and scalable EVs‐based nanocarriers for therapeutic and diagnostic applications.

## 2 Results and Discussion

### 2.1 EVs Characterization

Nanoparticle tracking analysis (NTA) was conducted to quantify particle concentration and size distribution across the three EVs preparations: naïve EVs (nEVs), control EVs samples (EVsCtrl), and osmotically lysed EVs (EVsLysed). As shown in Figure 1A, mean particle concentrations were 7 × 10^10^ ± 6 × 10^9^ particles/mL for nEVs, 1 × 10^10^ ± 3 × 10^9^ particles/mL for EVsCtrl, and 2 × 10^1^ ^1^ ± 5 × 10^10^ particles/mL for EVsLysed. These values correspond to a marked loss of particles in EVsCtrl and a statistically significant increase in EVsLysed compared to both nEVs (p = 0.005) and EVsCtrl (P ≤ 0.001). Both EVsCtrl and EVsLysed samples were subjected to filtration through Amicon 50 kDa centrifugal filters, a step known to deplete low‐molecular‐weight proteinaceous and colloidal components [63]. Studies have shown that such ultrafiltration devices can lead to substantial loss of proteins and small particles due to nonspecific adsorption or permeation through the filter membrane [63]. Therefore, the measured particle concentration likely underestimates the true content, particularly for vesicles or fragments under the 50 kDa threshold. Despite this, EVsLysed consistently exhibited a significantly higher particle count compared to both nEVs and EVsCtrl, reflecting the spontaneous formation of secondary vesicular structures together with improved visualization due to membrane disruption and cleaner sample preparation. The self‐assembly of phospholipid bilayers into vesicular structures is a well‐established thermodynamically favorable process, driven by the minimization of interfacial free energy [64, 65, 66, 67]. Upon rupture, lipid membranes naturally reconfigure into closed bilayer structures, forming new vesicles to reduce the energetic cost of exposing hydrophobic domains to aqueous environments.

**FIGURE 1:**
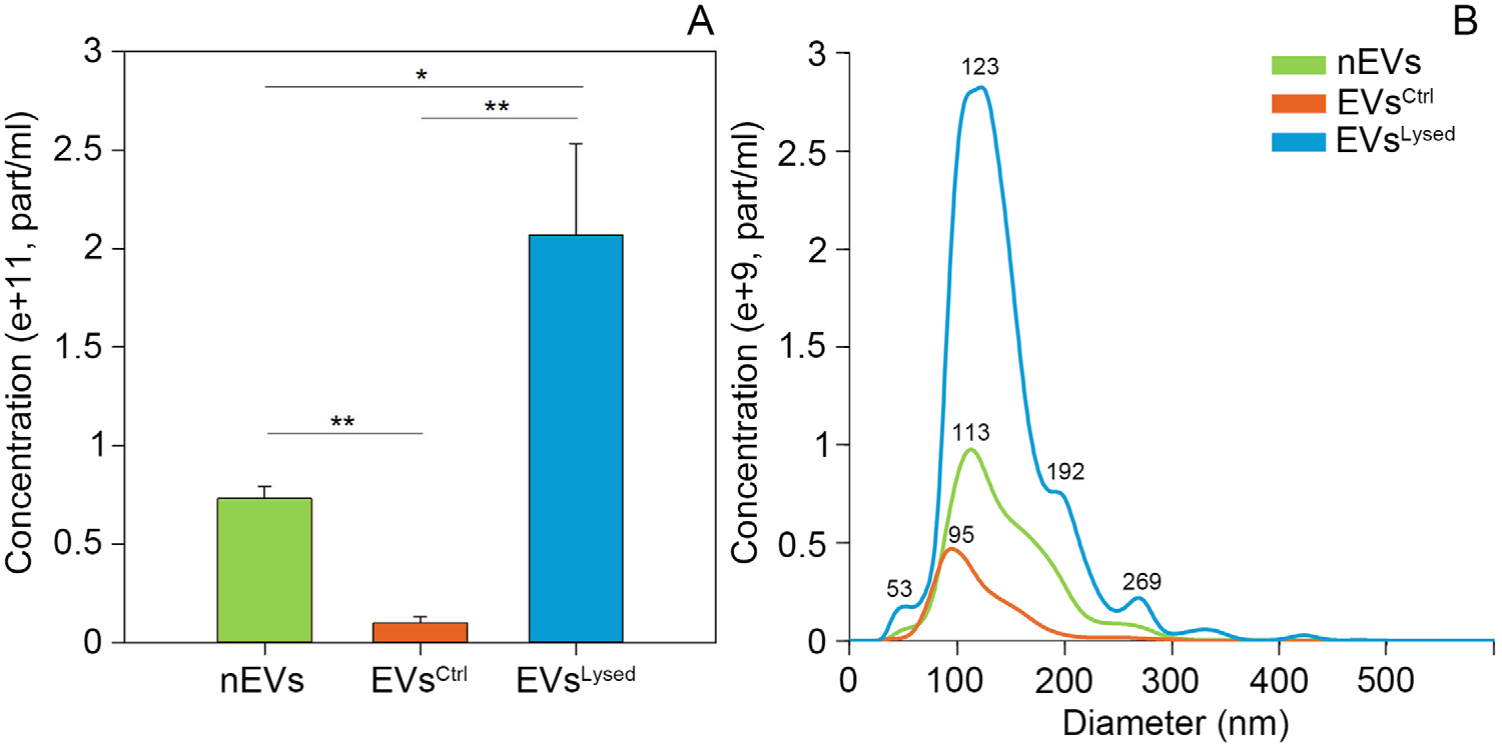
NTA concentration and distribution results. In panel A, results are represented as mean ± standard error on at least eight different experiments, with three technical replicates each. ^*^ *P* ≤ 0.05, ^**^ *P* ≤ 0.001 obtained with the *t*‐test. In panel B, a representative NTA distribution graph for each sample was displayed.

The corresponding size distribution profiles (Figure 1B) revealed asymmetrical, right‐skewed curves across all three conditions, with most particles measuring below 200 nm, consistent with the expected size range of small EVs. The nEVs population showed a distinct peak at 113 nm, while EVsCtrl exhibited a lower‐intensity and slightly left‐shifted peak at 95 nm, likely reflecting partial sample loss during handling and filtration. In contrast, EVsLysed displayed a sharper and more abundant peak at 123 nm, alongside additional minor populations at larger diameters (192 and 269 nm), and a small peak at ∼53 nm, possibly corresponding to nanoscale vesicular structures formed through incomplete resealing or high‐curvature membrane constriction after lysis. Importantly, although minor subpopulations were detectable, EVsLysed were consistently dominated by the 123 nm peak across independent preparations, and the overall size profile remained confined within the typical small EV range. All downstream functional assays were conducted on these unfractionated preparations and thus inherently reflect the integrated contribution of both the predominant small‐EV population and the minor size classes.

Consistent with this, total protein content assessed by the Bradford assay (Figure 2A) was significantly higher in nEVs (169 ± 14 µg/mL) compared to both 50 kDa filtered samples, with no significant difference between EVsCtrl (13 ± 2 µg/mL) and EVsLysed (16 ± 3 µg/mL), indicating that osmotic treatment does not increase protein recovery per se.

**FIGURE 2:**
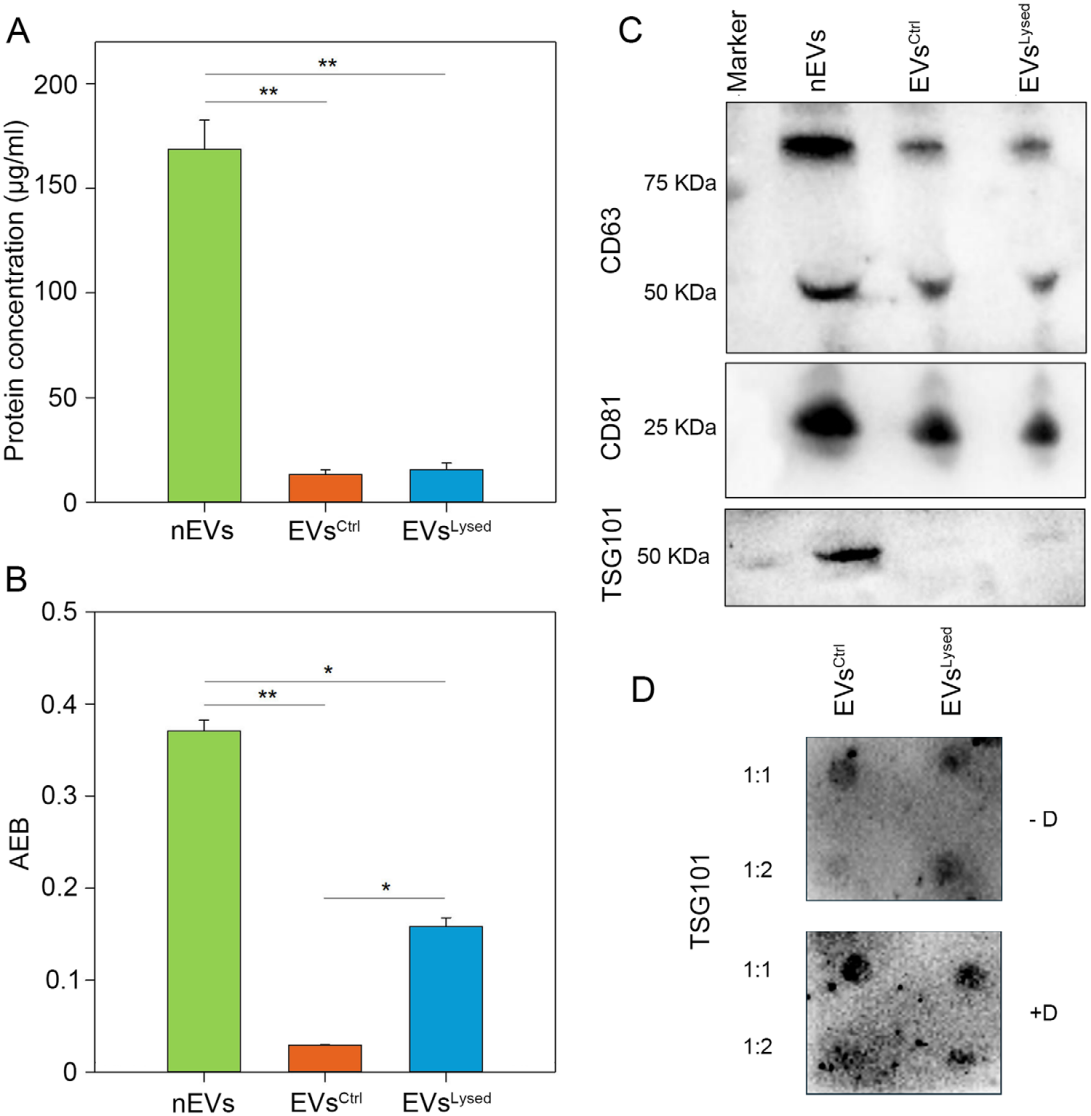
Protein analysis of nEVs, EVsCtrl, and EVsLysed. In panel A, the protein concentrations obtained through the Bradford assay of the three samples are reported; in panel B the SiMoA results, expressed as AEB, of the pantetraspanins are represented. Panel C depicts the Western blot for the CD63 and CD81 tetraspanins and TSG101, in D the dot blot of TSG101 performed on samples diluted 1:1 and 1:2 and tested in the absence (–D) and presence (+D) of Tween. Results are displayed as mean ± standard error on at least six different experiments for Bradford, and two replicates for SiMoA. ^*^ *P* ≤ 0.05, ^**^ *P* ≤ 0.001 obtained with the t‐test for Bradford and pantetraspanin.

The digital ELISA Single Molecule Array (SiMoA) assay results showed that the tetraspanin content (Figure 2B) was, as expected, more abundant on nEVs (p = 0.001 vs. EVsCtrl and p = 0.005 vs. EVsLysed), while between the two processed samples, tetraspanins were more abundant in the lysed sample (p = 0.006).

Western blot confirmed the presence of CD81 and CD63 in all vesicle preparations, while TSG101 was identified only in nEVs (Figure 2C). The negligible detection of TSG101 in both EVsCtrl and EVsLysed can be explained by the dilution and reconcentration steps required for their preparation. To verify this, dot blot analysis (Figure 2D) was performed. In the absence of Tween (–D), which prevents membrane permeabilization, immunorecognition of internal proteins such as TSG101 was hindered, resulting in only a faint signal, particularly in EVsCtrl. In contrast, in the presence of Tween (+D), comparable smeared signals were observed in both EVsCtrl and EVsLysed, supporting the idea that osmotic lysis promotes leakage and redistribution of TSG101 (and possibly other internal cargoes), making it partially detectable even without membrane permeabilization [68, 69].

To enable direct comparison between samples with different volumes and dilution factors, particularly given the 1:50 dilution imposed by hypotonic or physiologic incubation, EVsCtrl and EVsLysed were reconcentrated using 50 kDa ultrafiltration devices, a necessary step to standardize concentration prior to analysis. After normalization, nEVs showed the highest protein and tetraspanin content per vesicle (24.14 µg and 0.05 Average Enzyme per Bead (AEB) per 10^10^ particles, respectively), indicating a structurally and functionally intact population [70]. EVsCtrl displayed intermediate values, while EVsLysed exhibited only 0.80 µg of protein and 0.008 AEB of tetraspanins per 10^10^ particles. The membrane‐to‐total protein ratio was ∼4.5 fold higher in EVsLysed than in nEVs or EVsCtrl, supporting that hypotonic lysis promotes the release of intraluminal proteins and generates cargo‐depleted vesicles [71].

Electron microscopy was used to investigate the morphology of EVs derived from B lymphocytes under different treatment conditions (Figure 3). Panels A, D, and G correspond to transmission electron microscopy (TEM) images of nEVs; panels B, E, and H to TEM images of control EVs incubated in isotonic saline, while panels C, F, and I refer to TEM images of EVs lysed. nEVs appear numerous and well preserved, with electron‐dense membranes and uniform shape. In contrast, EVsCtrl and EVsLysed, both diluted 1:50 and concentrated using 50 kDa Amicon filters, show lower background compared to nEVs—likely due to sample loss during filtration. Notably, the EVsLysed sample shows a higher number of vesicles than EVsCtrl, despite undergoing identical processing steps. This increase is likely due to the osmotic lysis process, which may lead to fragmentation of larger vesicles followed by reassembly into smaller ones. Scanning electron microscopy (SEM) images (panels J–L) show nEVs, EVsCtrl, and EVsLysed samples, in which vesicles display the characteristic spherical or cup‐shaped morphology widely described in the literature, confirming their vesicular identity [72, 73]. nEVs (panel J) appear as well‐dispersed, rounded structures; EVsCtrl (panel K) display heterogeneous particle sizes with intact morphology; and EVsLysed (panel L) show vesicles with comparable structural features, without obvious signs of aggregation or degradation.

**FIGURE 3:**
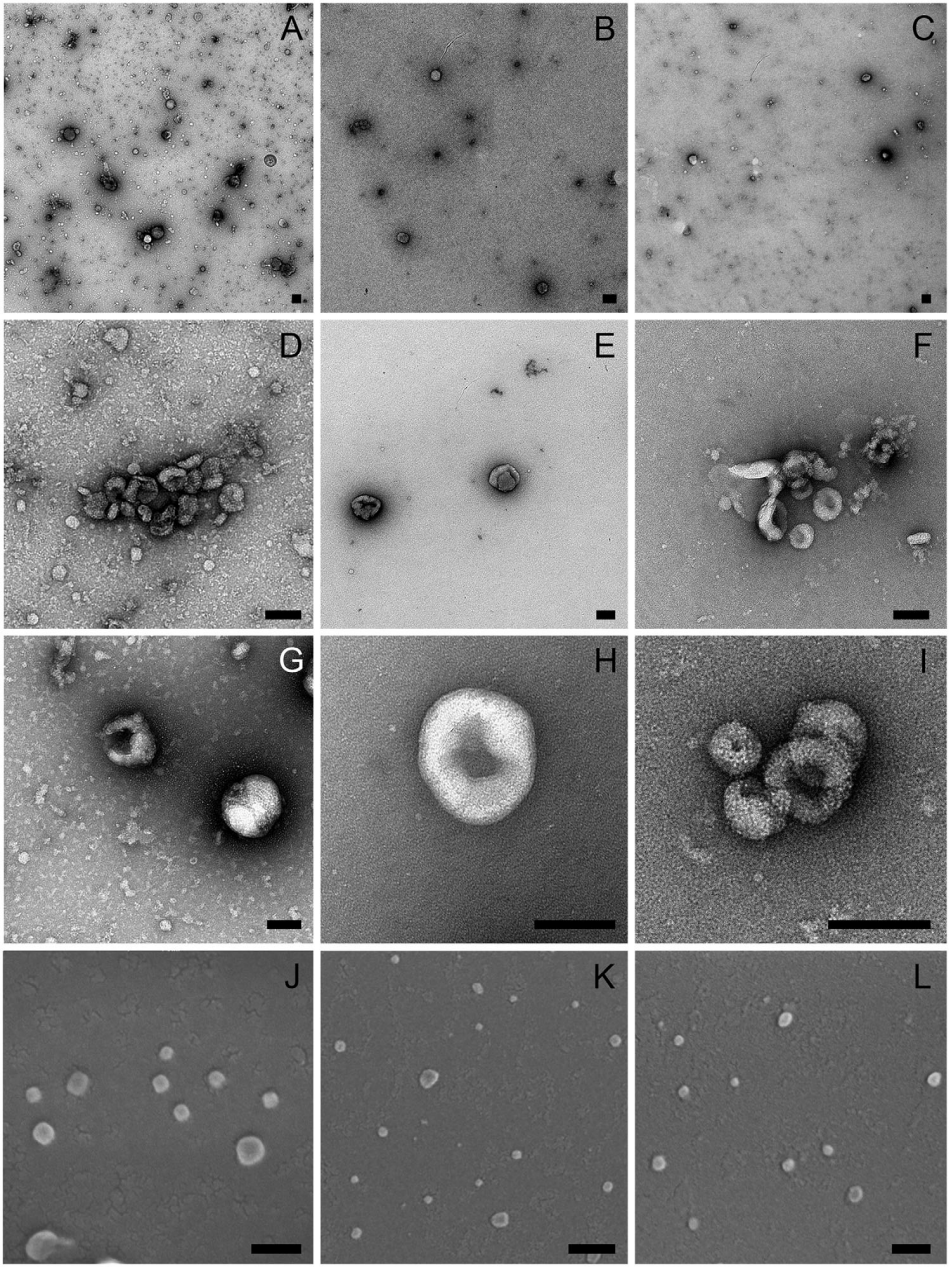
Ultrastructural analysis of EVs by electron microscopy. Panels from A to I represent transmission electron microscopy images where: A, D, and G were obtained from nEVs samples, B, E, and H from EVsCtrl, and C, F, and I from EVsLysed. Images J to L are scanning electron microscopy images of nEVs (J), EVsCtrl (K), and EVsLysed (L). Scale bars are 100 nm.

Single‐particle Atomic Force Microscopy (AFM) morphometry was employed to evaluate the size, abundance, and mechanical stiffness of EVs. The main results are summarized in Figure 4. All three samples were found to contain a large proportion of particles having morphological and mechanical characteristics compatible with those of intact EVs [74].

**FIGURE 4:**
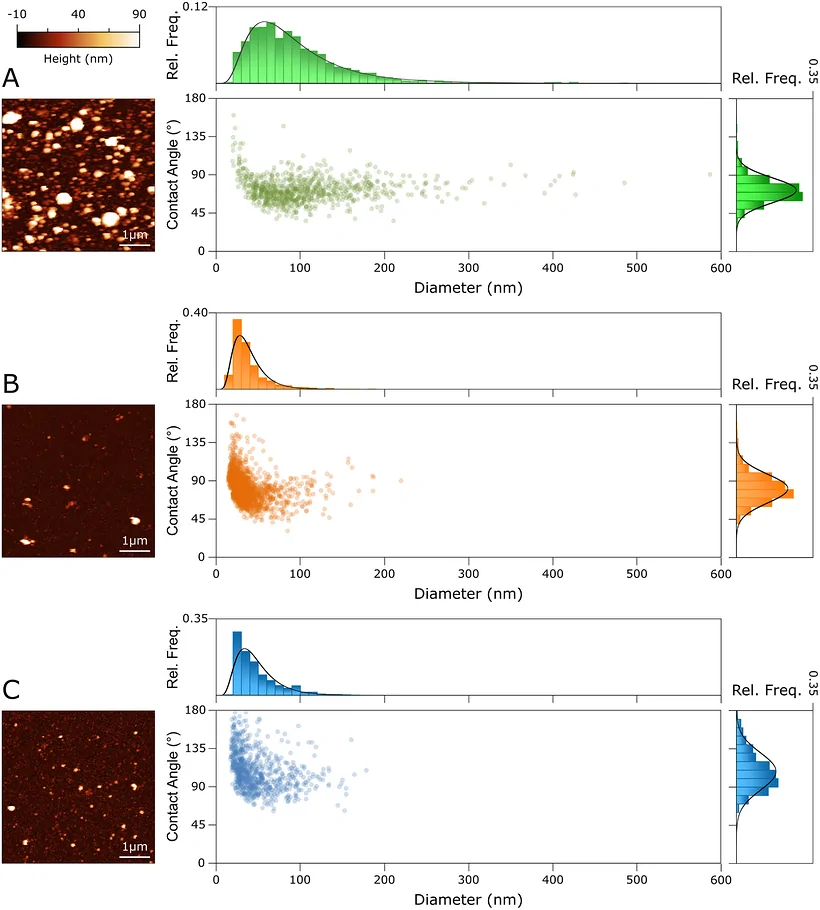
Atomic force microscopy imaging and quantitative morphometry for nEVs (A), EVsCtrl (B), and EVsLysed (C). Left: representative liquid AFM micrographs (all images share the same lateral dimensions and false color vertical scale). Right: surface CA vs solution Diameter (sD) scatterplots of ∼800 individual particles found in each of the samples. Scatterplots are accompanied by CA (right) and sD (above) relative frequency histograms, including normal (CA) or log‐normal (sD) distribution fits (solid black curves).

All EV size distributions were found to be log‐normal; EVsCtrl and EVsLysed showed very similar size distributions with the vast majority of particles having diameters < 100 nm, while nEVs were found to have a considerably larger size variance with a significant proportion of EVs in the 100–500 nm diameter range. Conversely, the Contact Angle (CA) distributions of nEVs and EVsCtrl were almost superimposable and centered at considerably lower values than that of EVsLysed (P ≤ 0.0001).

Since aliquots of the same volume from all samples were deposited following the same protocol (see materials and methods), it is possible to use particle surface densities as measured via AFM images as a rough estimate of the relative concentrations of samples [75]. Following this procedure, EVsCtrl were found to be ∼10 times less concentrated than the other two samples.

To investigate the lipid remodeling induced by the vesicle treatment, an untargeted lipidomic analysis was performed to quantitatively compare the lipid profiles of nEVs and EVsLysed. This analysis allowed the identification and quantification of 431 lipid species, divided into 23 distinct lipid classes: cholesteryl esters (CE), ceramides (Cer), cholesterol, diacylglycerols (DG), hexosylceramides (HexCer), lysophosphatidylcholines (LPC), ether‐linked lysophosphatidylcholines (LPC‐O), lysophosphatidylethanolamines (LPE), N‐acylethanolamines (NAE), phosphatidylcholines (PC), ether‐linked phosphatidylcholines (PC‐O), phosphatidylethanolamines (PE), ether‐linked phosphatidylethanolamines (PE‐O), plasmalogen phosphatidylethanolamines (PE‐P), phosphatidylglycerols (PG), phosphatidylinositols (PI), phosphatidylserines (PS), sulfatides (SHexCer), sulfonolipids (SL), sphingomyelins (SM), sphinganines (SPB), sterols (ST), and triglycerides (TG).

Among the identified lipid species, 49 were significantly enriched and 11 decreased in EVsLysed compared to nEVs (p < 0.05; fold‐change > 1.3) (Figure 5A). Principal component analysis (Figure 5B) and partial least squares discriminant analysis (Figure 5C) showed a clear separation between the two vesicle types. Figure 5A,D depict the significant enrichment in medium‐ and long chain fatty acid‐PS (PS 36:4, PS 34:1; PS 36:2), typical SMs, plasmalogen phospholipids (mainly PE‐O species), and polyunsaturated LPE (22:6, 22:5), with a concomitant decrease of saturated FA (16:0, 18:0), DG (32:0–36:0), and LPCs (16:0 and 18:1). PC supports curvature and stability, and its hydrolysis produces LPC, a pro‐inflammatory signaling lipid [76, 77]. Ether‐linked phospholipids (PC‐O and PE‐O) improve membrane fluidity and oxidative resistance [78, 79]. SPH enhances rigidity and contributes to EV–target cell interactions; upon processing, it generates ceramides, important in EV biogenesis [80]. LPE influences membrane curvature and signaling [81]. Both plasmalogens and SPH are lipid raft components, suggesting that these microdomains are preserved or even enriched in EVsLysed [82]. In contrast, the reduction of cargo‐associated DG, FA, LPC, and specific TGs supports loss of luminal content during lysis, further accentuated by 50 kDa ultrafiltration, which removes small, unbound lipids [83].

**FIGURE 5:**
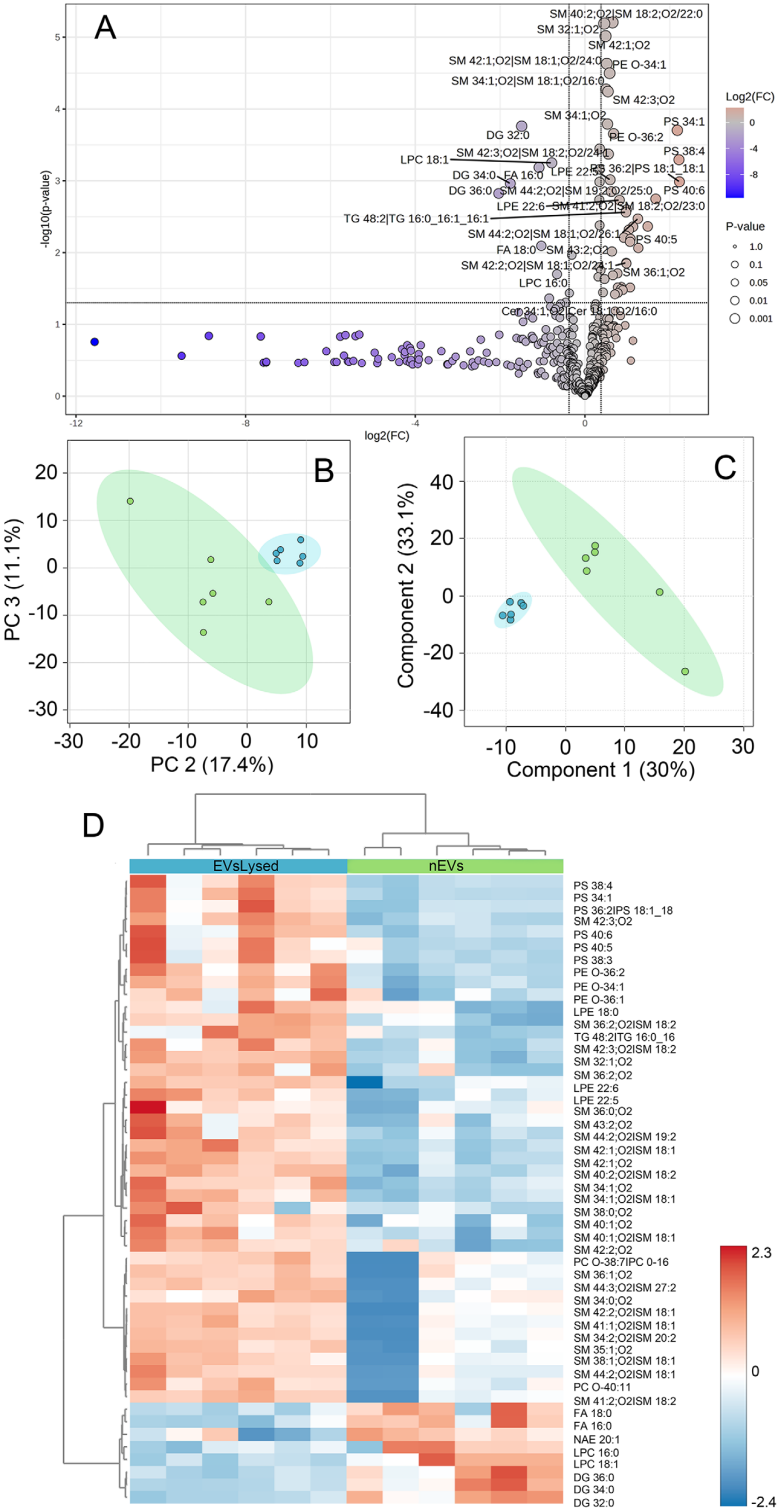
Multivariate and univariate analyses comparing EVsLysed vs. nEVs. (A) Volcano plot showing up‐ and down‐regulated lipid species (p‐value < 0.05, fold change (FC) > 1.3). (B) Principal Component Analysis (PCA) score plot. (C) Partial Least Squares Discriminant Analysis (PLS‐DA). (D) Hierarchical Heatmap showing up‐ and down‐regulated lipid species. In (B, C, D), Green is for nEVs while blue is for EVsLysed.

LPC reduction is notable given its role as a pro‐inflammatory mediator [84]. DG are bioactive lipids regulating budding and fusion [85], while FA act as energy reservoirs and modulators of vesicle–cell interactions. Ceramides stiffen membranes, drive EVs biogenesis, and mediate signaling [86]. Altogether, enrichment of structural phospholipids (PS, PC, plasmalogens, SPH, LPE) and depletion of cargo lipids (DG, FA, LPC, selected TGs) indicate that lysis preserves the vesicle envelope while removing internal material.

At the lipid class level, PS were the most up‐regulated in EVsLysed, followed by SM, PC (diacyl‐ and plasmalogen), PE plasmalogen, and LPE, while DG, FA, and LPC showed significant down‐regulation (Figure 6A,B). Although TG as a class did not vary significantly, the species TG 48:2 was enriched while TG 14:0_16:0_22:5 was significantly reduced, suggesting selective retention or loss of intraluminal cargo.

**FIGURE 6:**
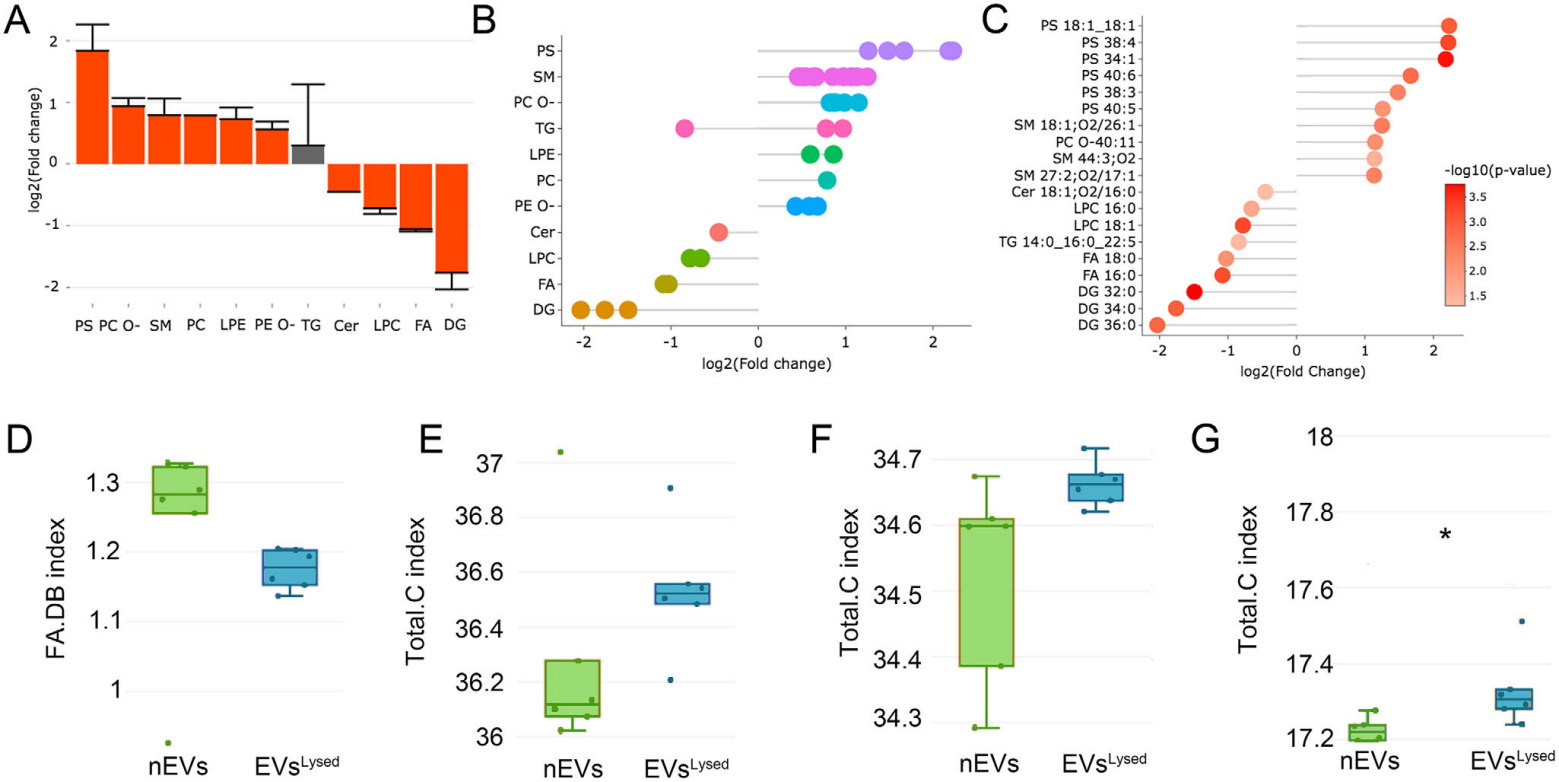
Differential analysis of lipid species comparing EVsLysed vs nEVs. (A) Bar chart displaying up‐ and down‐regulated lipid classes (>1.3 FC). (B) Lollipop chart showing the regulation of lipid classes and their distribution among different species. (C) Lollipop chart highlighting the most significantly up‐ and down‐regulated lipid species. (D) Average number of double bonds in fatty acids. (E) Average chain length of phosphatidylcholines. (F) Extended range of chain lengths for phosphatidylcholines. (G) Average chain length of lysophosphatidylcholines. All comparisons were performed using a threshold of fold‐change > 1.3 and *p* < 0.05. ^*^<0.05; ^**^<0.01; ^***^<0.001. The grey bar in panel A indicates a non‐significant modulation of TG lipid class.

Detailed modulation of individual species (Figure 6C) confirmed increases in PS 18:1_18:1, PS 38:4, PS 34:1, and PS 40:6, and decreases in DG 36:4, DG 34:0, DG 32:0, FA 16:0, and FA 18:0. Structural analysis showed that the average number of double bonds was higher in nEVs, indicating greater unsaturation, while chain length was longer in EVsLysed (Figure 6D–G). This compositional shift points to a more ordered bilayer in EVsLysed, consistent with AFM evidence of increased stiffness and reduced size. The lipidomic profiling was performed on two independent biological EV preparations and should therefore be regarded as exploratory. While the observed lipid rearrangements consistently support the membrane remodeling process, broader biological replication will be required in future studies to fully capture inter‐batch variability.

To investigate the impact of osmotic lysis on the vesicle proteome, an untargeted proteomic approach was employed to quantify and compare protein content in nEVs and EVsLysed samples across three independent biological replicates. Proteomic analysis identified and quantified 1655 proteins: hierarchical clustering heatmap and partial least square analysis (Figure 7A,B) clearly showed that nEVs and EVsLysed vesicles are characterized by a different proteome. The analysis of the enriched cellular components of the total identified proteins from the EVsLysed sample revealed a prominent enrichment in both cytoplasmic and exosomal proteins, which accounted for 61.8% and 50.4% of the mapped proteins, respectively (Figure 7C). These results indicate that the protein content of lysed EVs is largely associated with both vesicular and cytosolic compartments. Consistent with the lipidomic data, proteomic results further confirmed that EVsLysed differ from naïve EVs in several specific aspects. In particular, the levels of 641 proteins were statistically different when comparing EVsLysed and nEVs vesicles, with 587 enriched proteins and 54 decreased proteins in EVsLysed vesicles (*p*‐value: 0.05; Fold‐change: 1.3). It is interesting to note that, among the 587 proteins increased in EVsLysed vesicles, 129 are indicated as transmembrane proteins (Figure 7D), underlining an enrichment and remodelling of this class of proteins in EVsLysed vesicles. Proteome changes are also highlighted in the Volcano plot (Figure 7E): the graph shows increased (red dots) and decreased (blue dots) proteins for the comparison between EVsLysed and nEVs: selected highly modulated and relevant proteins were reported in the plot.

**FIGURE 7:**
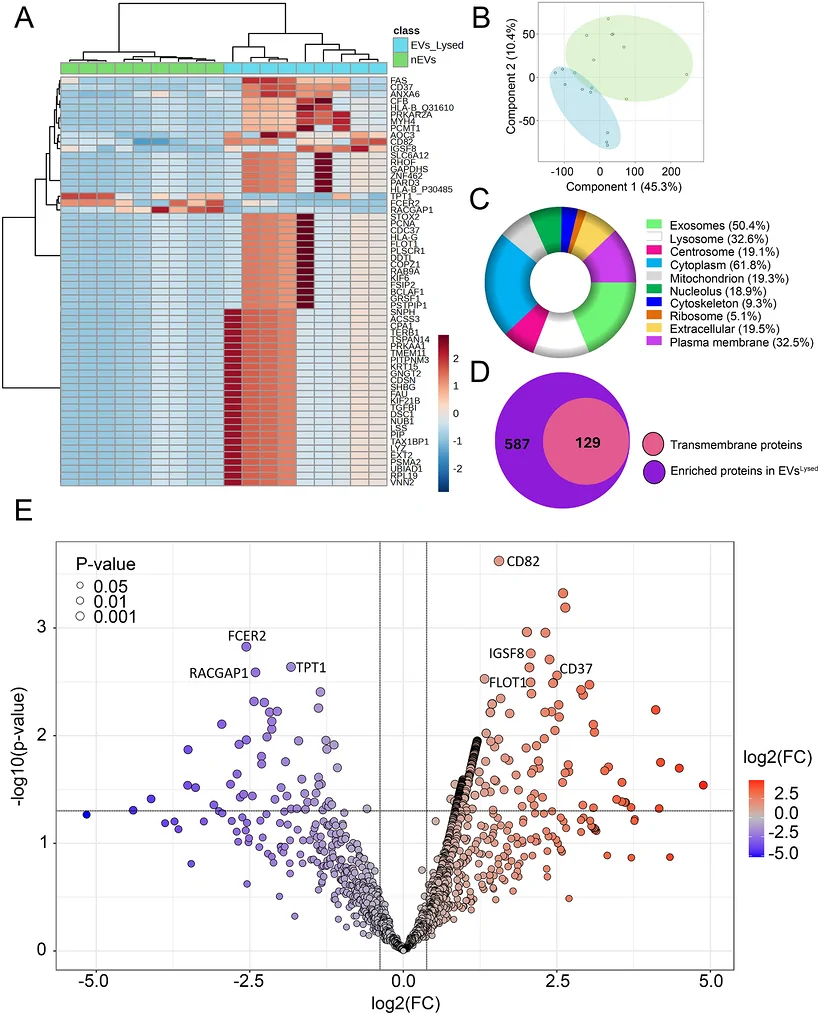
Proteomic analysis EVsLysed and nEVs groups. (A) Hierarchical clustering heatmap of the quantified proteins’ abundances of nEVs (green) and EVsLysed (light blue) samples. (B) Partial least squares analysis between the quantified proteins of nEVs (green) and EVsLysed (light blue). (C) Cell component enrichment of the total identified proteins: representation of the first 10 most significant cell components. (D) Overlapping between increased proteins in EVsLysed (purple) and Transmembrane proteins according to Uniprot (pink). (E) Volcano‐plot of EVsLysed in comparison with nEVs (Fold‐change: 1.3; *p*‐value: 0.05).

In order to characterize the biomolecular features of EVsLysed compared to nEVs, canonical pathway analysis was performed using Ingenuity Pathway Analysis (IPA) on significantly modulated proteins (p‐value < 0.05; fold‐change > 1.3). The bar plot in Figure 8A highlights the most relevant pathways ranked by activation Z‐score. A Z‐score > 2 indicates predicted activation (shown in red), whereas a Z‐score < −2 indicates predicted inhibition, as observed for the BAG2 signaling pathway. Pathways with no predicted activation state, such as RHOGDI signaling and Integrin signaling, are shown in grey.

**FIGURE 8:**
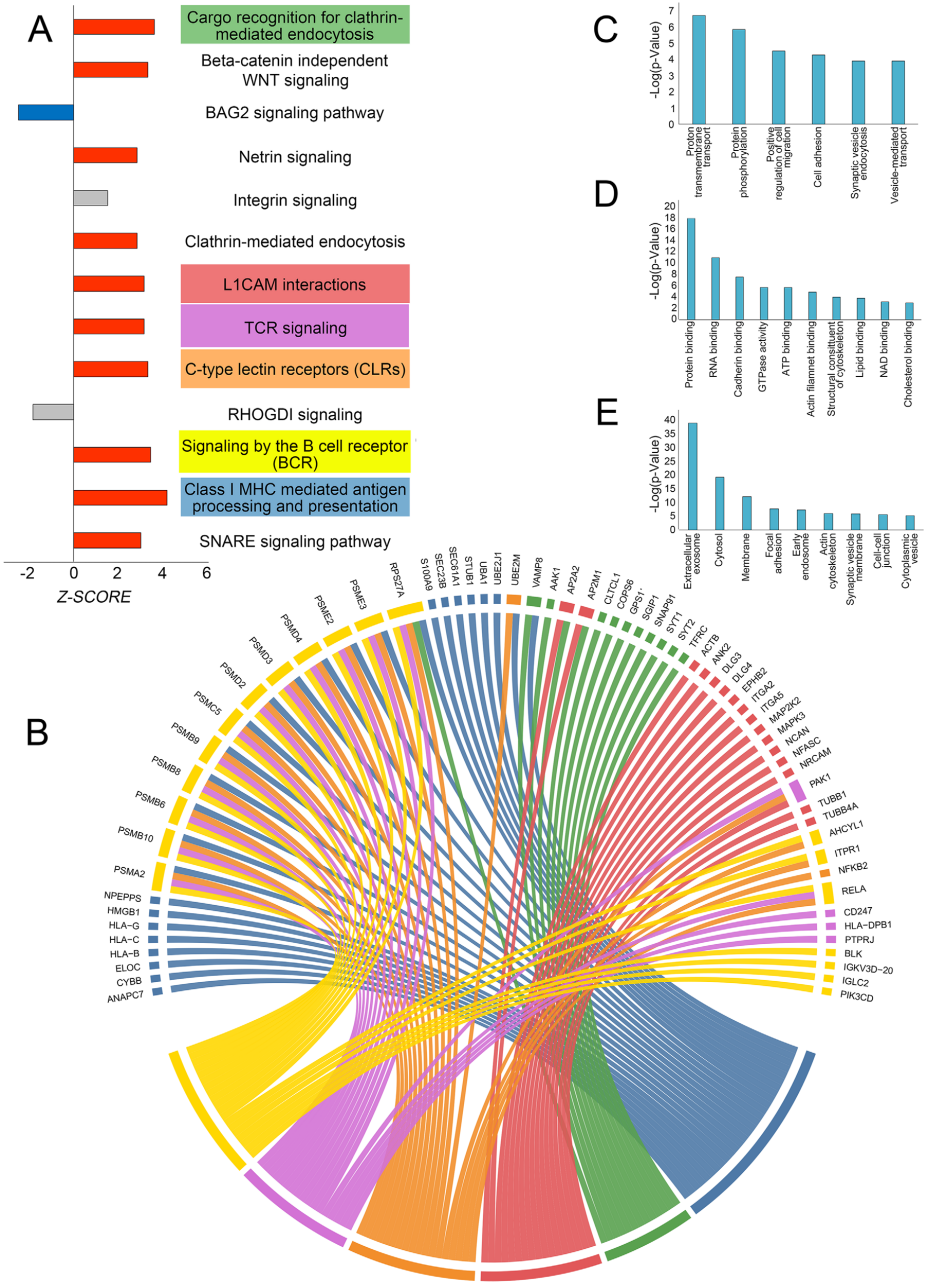
Proteomic analysis EVsLysed and nEVs groups. (A) Selected canonical pathways from the IPA of modulated proteins (*p*‐value: 0.05; Fold‐change: 1.3): the bar plot reports some of the most significant canonical pathways as a function of the z‐score. A Z‐score >2 indicates a prediction of activation (red); a Z‐score←2 indicates a prediction of inhibition (blue); not predicted are reported in grey. (B) CORD plot illustrating six representative canonical pathways from panel a and their associated proteins. Each color corresponds to a specific pathway: green for “cargo recognition for clathrin‐mediated endocytosis”, red for “L1CAM interactions”, violet for “TCR signaling”, orange for “C‐type lectin receptor signaling”, yellow for signaling B cell receptor”, and blue for “class I MHC‐mediated antigen processing and presentation”. (C–E) Bar plots showing the most significantly enriched GO terms in EVsLysed, including biological processes (C), molecular functions (D), and cellular components (E), ranked by –log(p‐value).

Following this analysis, the Chord Plot in Figure 8B illustrates the network of proteins contributing to the six top‐ranked pathways identified in EVsLysed. This panel reveals a dense cluster of functionally interconnected proteins, primarily related to the ubiquitin–proteasome system. Several proteasome subunits were prominently enriched, including PSMA2, PSMB6, PSMB8, and PSMB10 from the 20S core, as well as regulatory components such as PSMC5, PSMD2, PSMD3, and PSMD4 from the 19S cap. Additional contributors included PSME2 and PSME3, which serve as alternative proteasome activators [87], RPS27A, an ubiquitin‐fused ribosomal protein [88], and PAK1, a kinase involved in cytoskeletal remodeling and vesicle dynamics [89].

These proteins exhibited strong overlap with multiple signaling cascades that emerged as significantly enriched in the pathway analysis, including cargo recognition for clathrin‐mediated endocytosis, L1CAM interactions, T cell receptor (TCR) signaling, C‐type lectin receptor (CLR) pathways, class I MHC‐mediated antigen processing and presentation, and B cell receptor (BCR) signaling. Many of these pathways are closely associated with immune regulation and vesicle‐mediated trafficking [89, 90]. The connectivity visualized in the Chord Plot suggests that EVsLysed carry a selective and coordinated proteome, potentially involved in intracellular trafficking, immune modulation, and proteostasis.

In parallel, functional annotation clustering of the enriched proteins in EVsLysed was performed using DAVID software (Figure 8C–E). Gene Ontology (GO) analysis revealed a strong enrichment of terms related to membrane‐associated functions, protein and receptor binding, and vesicle‐mediated processes. Among the Biological Process categories, highly significant annotations included cell adhesion, synaptic vesicle endocytosis, and vesicle‐mediated transport, suggesting a close relationship between the identified proteome and intracellular trafficking mechanisms. Regarding Molecular Function, enriched terms included protein binding, cadherin binding, and cholesterol binding, further highlighting the involvement of these proteins in membrane dynamics and intercellular communication.

As for Cellular Component, the most significantly represented categories were extracellular exosomes, membrane, vesicles, focal adhesion, cytosol, early endosome, and actin cytoskeleton, reflecting the subcellular origin and dynamic nature of the proteins recovered in EVsLysed [91]. These findings align with the pathway and network‐based results described in panels a and b, and reinforce the notion that the lumen of EVsLysed is enriched in proteins involved in cell signaling, immune regulation, membrane trafficking, and cytoskeletal organization, hallmark features of vesicles with active roles in intercellular communication [92].

### 2.2 Effects of the Osmotic Lysis on Vesicular Cargo

To evaluate the potential of osmotic lysis for engineering extracellular vesicles, we investigated both their ability to release native cargo and their capacity to incorporate exogenous material. As a proof‐of‐concept to test whether the lysis process could simultaneously favor the loading of external agents, we selected gold nanoparticles (AuNPs) as a model inorganic cargo. TEM revealed a marked interaction between AuNPs and EVsLysed membranes (Figure 9A,B), with AuNPs appearing closely associated with vesicular structures. In contrast, EVsCtrl samples showed minimal interaction (Figure 9C,D), with nanoparticles remaining largely unbound in the background. The observed difference might also reflect differences in surface accessibility, local curvature, or protein corona composition induced by the lysis process, rather than a complete lack of affinity in intact vesicles. Further mechanistic studies would be required to dissect these effects.

**FIGURE 9:**
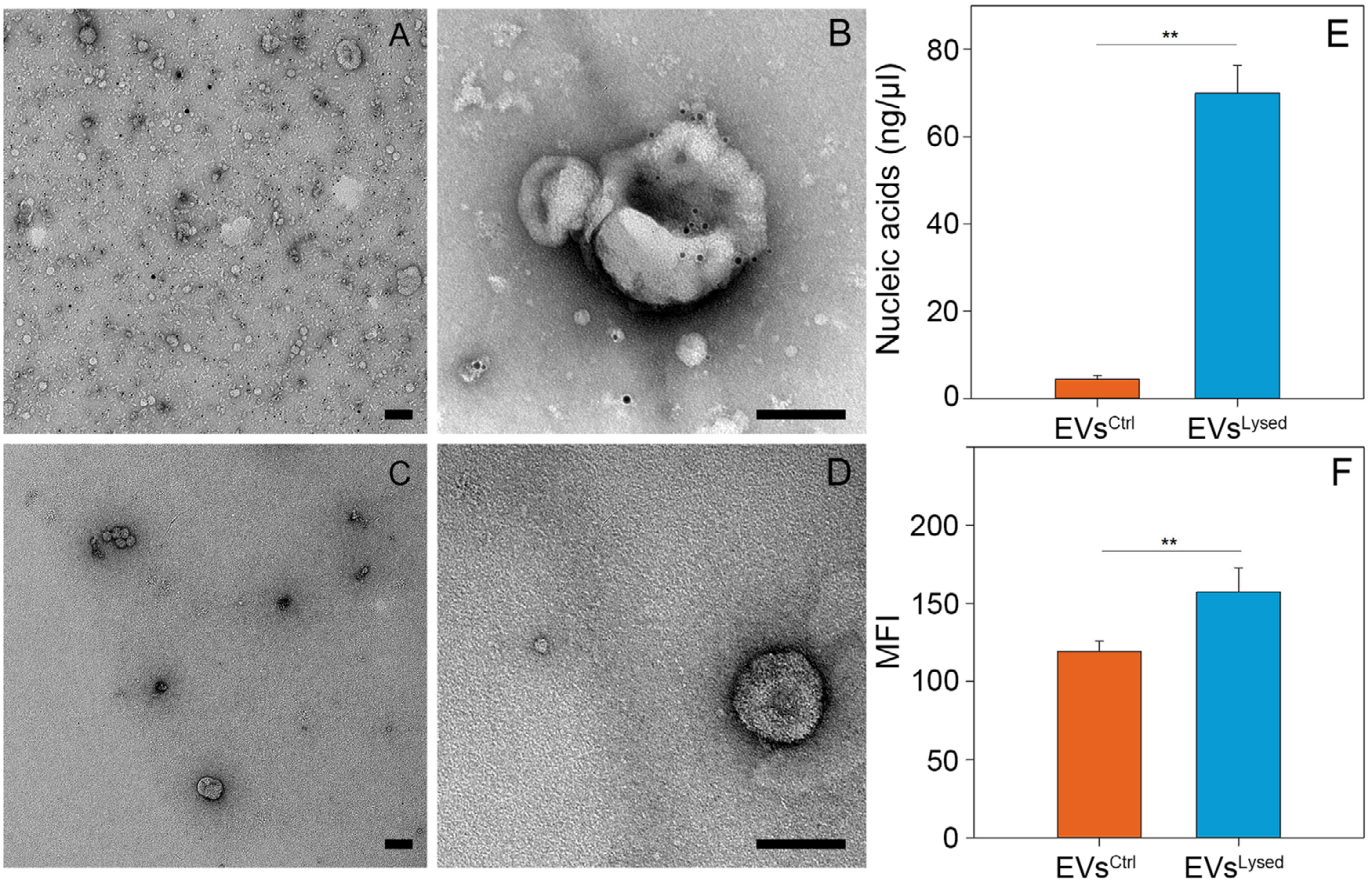
EVsCtrl and EVsLysed native cargo release and customized loading. Panels A and B are the TEM images of EVsLysed loaded with AuNPs, panels C and D of EVsCtrl loaded with AuNPs, scale bars are 100 nm. Graph E is the result of the nucleic acid content, and Graph F is the cytofluorimetric analysis of the internalization of AuNPs in EVsCtrl and EVsLysed. Comparisons are made with *t*‐test analysis, and ^**^ = P ≤ 0.001 are considered significant.

To assess the release of native vesicular cargo, we measured the nucleic acid content in the supernatants collected after sample processing. As shown in Figure 9E, EVsLysed released significantly more nucleic acids than EVsCtrl (P ≤ 0.001), confirming a more extensive loss of internal components following membrane disruption. The supernatants analyzed were obtained after centrifugation of EVs suspensions post‐lysis (or mock treatment), and reflect the fraction of nucleic acids not retained within vesicular structures. These results are consistent with the evidence from our protein analysis (see section on vesicular proteins’ characterization), where the loss of intraluminal markers such as TSG101 indicated membrane rupture, further validating that osmotic lysis efficiently promotes cargo depletion. It is important to emphasize that osmotic remodeling predominantly acts on luminal EV cargo and co‐isolated soluble contaminants, while preserving the membrane scaffold and allowing the formation or reorganization of protein coronas that can contribute to targeting, binding, and uptake processes at the interface with recipient cells. In this framework, the remodeled vesicles are designed as “detoxified” membrane‐derived carriers, in which potentially harmful endogenous intraluminal signals are largely depleted, whereas surface and corona‐associated components can still support specific interactions with recipient cells, making them suitable candidates for tunable drug delivery applications. Thanks to this combination of a largely emptied interior and a biologically competent surface, remodeled EVs can, in principle, operate as Trojan horse‐like carriers, exploiting their native or reshaped molecular interfaces to gain access to target cells while transporting a controlled exogenous payload. At the same time, membrane‐associated molecules and newly formed corona layers may retain oncogenic or immunomodulatory properties in a source‐ and indication‐dependent manner. The present work therefore reduces, but does not eliminate, potential safety concerns, and a comprehensive assessment of membrane‐ and corona‐associated risk factors will require dedicated in vitro and in vivo immunological and oncological studies in appropriately defined disease models.

Flow cytometry using fluorescently labeled AuNPs (Figure 9F) confirmed significantly greater association with EVsLysed compared to EVsCtrl (P ≤ 0.001), as reflected by higher mean fluorescence intensity (MFI). While this assay does not discriminate between surface binding and internalization, the combined imaging and fluorescence data indicate that membrane remodeling enhances vesicle–nanoparticle interactions.

The stronger association observed in our experiments between AuNPs and EVsLysed compared to control samples further supports the hypothesis that hypo‐osmotic treatment alters membrane composition and structure, enhancing the vesicles’ loading capacity. Importantly, following filtration through Amicon 50 kDa devices, AuNPs remained predominantly associated with the lysed vesicles, validating the potential of this protocol for incorporating small molecules, drugs, or nanoparticles into engineered EVs.

These results align with extensive literature describing how nanoparticle–bilayer interactions depend on membrane composition, curvature, and environmental factors such as ionic strength and protein corona formation [93, 94, 95, 96]. Moreover, our findings are consistent with previous studies demonstrating that AuNPs spontaneously interact with natural and synthetic lipid membranes, forming clusters [97, 98, 99]. Together, these findings reinforce the notion that osmotically lysed EVs represent a distinct and highly functional nanovesicle population with favorable characteristics for both diagnostic and therapeutic applications.

### 2.3 Differential Uptake of EVs Across Cell Types and Engineering States

To provide a direct validation of the proposed method for EVs engineering, we designed uptake assays aimed at testing both efficacy and selectivity. EVsCtrl and EVsLysed were compared in two complementary models: primary‐like B lymphocytes, from which the naïve vesicles were derived, and their malignant counterpart, Burkitt's lymphoma–derived Raji cells. This in vitro system is particularly powerful because it exploits vesicles released by circulating and ubiquitous immune cells, while enabling a direct comparison between healthy and tumor contexts.

EVsCtrl and EVsLysed were tested at two different concentrations, 5 and 10 µg/mL, and results were collected after three different times of treatment, two short‐term times (2 and 4 h) and a longer one (24 h). Results were analyzed with a three‐way analysis of variance (ANOVA) and represented in Figure 10.

**FIGURE 10:**
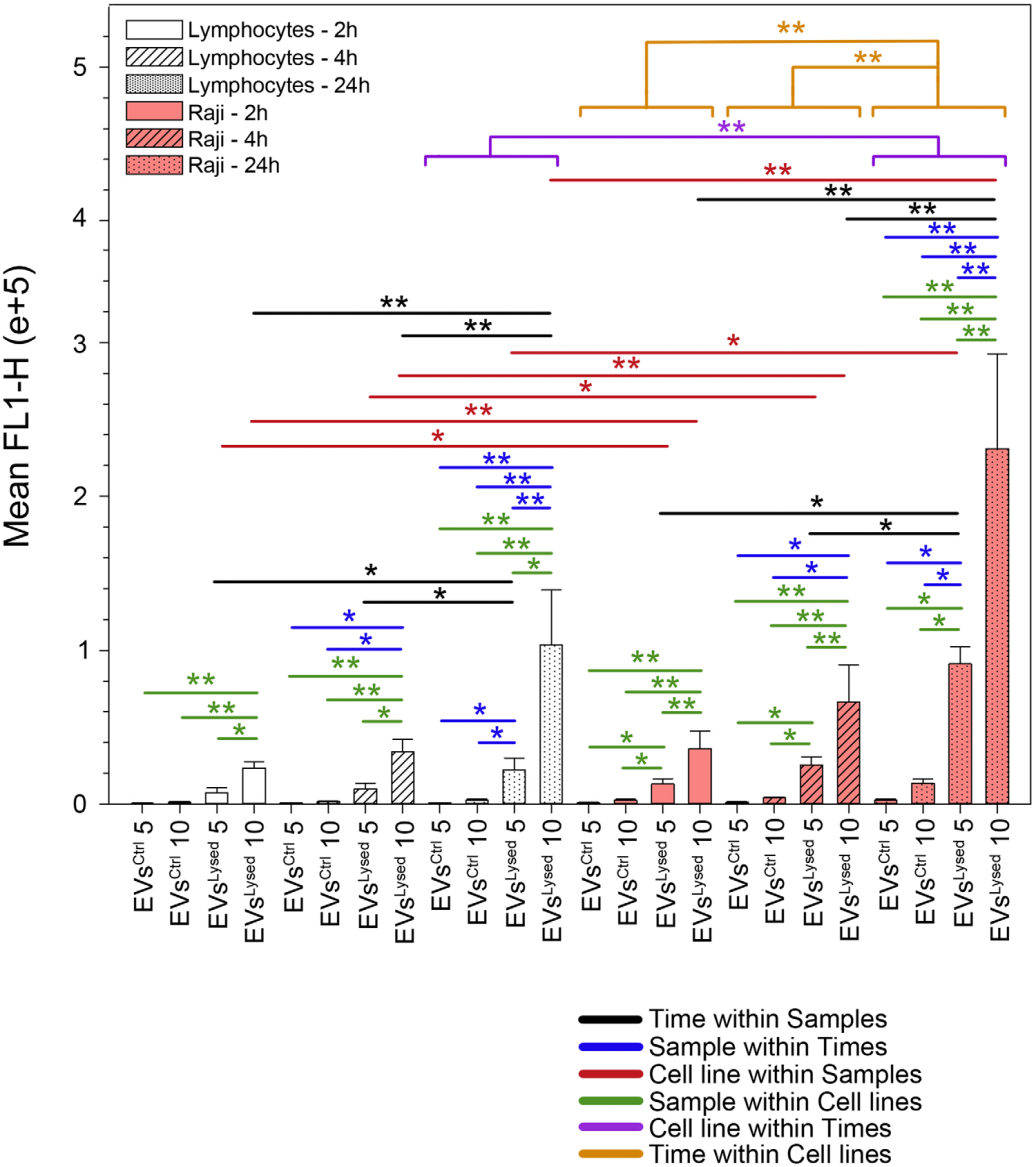
EVsCtrl and EVsLysed uptake results in B lymphocytes and Raji cell lines. In the figure, the internalization results of EVsCtrl and EVsLysed in B lymphocytes and Raji cell lines after 2, 4, and 24 h of treatment are represented. In detail, results for B lymphocytes are displayed in white and for Raji in red, while, regarding the treatment times, the 2 h are represented in solid color bars, the 4 h in dashed bars, and the 24 h of treatment in dotted bars. 5 and 10 are the two doses in µg/mL. Results are displayed as mean ± standard error (n = 3). Statistical analysis was carried out using a three‐way ANOVA and the *p*‐values of the significant comparisons are reported on the graph: Time within Samples in black, Sample within Times in blue, Cell line within Samples in red, Sample within Cell lines in green, Cell line within Times in violet, Time within Cell lines in orange. ^**^ = *P* ≤ 0.001 and ^*^ = *p* < 0.05 are considered significant.

When comparing uptake across the different cell lines, a statistically significant difference emerged between Raji cells and lymphocytes (*P* ≤ 0.001). However, when this result is examined in relation to the type of sample, the difference remains significant only for EVsLysed (p = 0.026 at 5 µg/mL and *P* ≤ 0.001 at 10 µg/mL). Similarly, the interaction between cell lines and incubation time revealed that a significantly higher internalization in Raji cells, compared to lymphocytes, was observed only after 24 h (*P* ≤ 0.001). In lymphocytes, there were no significant differences among the treatment times, while in Raji cells, the internalization increased after 24 h (*P* ≤ 0.001 vs. both 2 and 4 h).

After 2 h there were no significant differences between samples, but after 4 h EVsLysed at 10 µg/mL started to be internalized more than the two controls (p = 0.020 vs. EVsCtrl 5 µg/mL and p = 0.024 vs. EVsCtrl 10 µg/mL). After 24 h, both EVsLysed samples were internalized more than the controls (P ≤ 0.001 for EVsLysed 10 µg/mL vs. EVsCtrl 5 µg/mL and EVsLysed 10 µg/mL vs. EVsCtrl 10 µg/mL; p = 0.004 for EVsLysed 5 µg/mL vs. EVsCtrl 5 µg/mL and p = 0.008 for EVsLysed 5 µg/mL vs. EVsCtrl 10 µg/mL). There was also a difference between the two EVsLysed concentrations (P ≤ 0.001 EVsLysed 10 µg/mL vs. EVsLysed 5 µg/mL).

Generally, uptake of control samples was not influenced by time and remained constant, while the internalization of lysed samples depended on time, with significant differences between shorter and longer incubations (p = 0.017 and p = 0.036 for EVsLysed 5 µg/mL at 2 vs. 24 h and 4 vs. 24 h, respectively; P ≤ 0.001 for EVsLysed 10 µg/mL at 2 vs. 24 h and 4 vs. 24 h, respectively).

When comparing the different samples, EVsLysed at 10 µg/mL were internalized significantly more than all other samples (P ≤ 0.001). Even at the lower concentration, EVsLysed showed significantly higher uptake than EVsCtrl (p = 0.016 vs. EVsCtrl at 5 µg/mL and p = 0.025 vs. EVsCtrl at 10 µg/mL). Considering differences between samples across the two cell lines, EVsLysed were internalized significantly more in Raji cells than in B lymphocytes. Specifically, EVsLysed at 10 µg/mL were taken up more than both control samples in both B lymphocytes (p = 0.001) and Raji cells (P ≤ 0.001). At 5 µg/mL, uptake in lymphocytes was comparable to that of the control samples, while in Raji cells, internalization remained significantly different from EVsCtrl regardless of concentration (p = 0.008 vs. EVsCtrl at 5 µg/mL and p = 0.015 vs. EVsCtrl at 10 µg/mL). Comparing the two doses of EVsLysed, uptake was dose‐dependent in both B lymphocytes and Raji cells, with higher concentrations leading to greater internalization (p = 0.013 for lymphocytes; P ≤ 0.001 for Raji).

Beyond the quantitative assays, our proteomic analysis provided mechanistic insights into the altered behavior of EVsLysed. The enhanced tumor cell uptake of remodeled EVs is therefore interpreted on the basis of correlative membrane compositional changes, including phosphatidylserine exposure and enrichment of membrane‐associated proteins, and the precise molecular mechanisms mediating this selectivity remain to be formally elucidated in future receptor‐blocking and pathway‐specific studies. The preferential uptake of lysed vesicles by Raji cells over healthy lymphocytes correlated with the enrichment of proteins mediating vesicle–cell interactions. Notably, CD82, a tetraspanin involved in microdomain organization, and hyaluronan and proteoglycan link protein 1, which facilitates binding to hyaluronic acid, were upregulated [100, 101]. Together with CD37, associated with leukocyte recruitment, these molecules highlight an enhanced propensity of EVsLysed to engage with recipient cells. It is important to note that this ‘enrichment’ is defined relative to naïve EVs after normalization and reflects a redistribution of the original protein complement following selective loss of other components, rather than the de novo appearance of entirely new membrane species. In this sense, osmotic remodeling reshapes the relative representation of pre‐existing membrane‐ and raft‐associated proteins within the vesicle population, consistent with the broader lipid and protein remodeling observed, without implying that all other membrane determinants are preserved unchanged. In parallel, enrichment of pathways such as clathrin‐mediated endocytosis, L1CAM interactions, and C‐type lectin receptor signalling [102] suggests multiple routes for internalization.

Interestingly, our dataset also revealed the presence of HLA class I molecules (e.g., HLA‐B and HLA‐C), pointing to an immunological dimension. Gene ontology analysis further confirmed the remodeling of EVsLysed, with significant enrichment in processes linked to endocytosis, vesicle‐mediated transport, and adhesion. Overrepresented molecular functions such as protein and cholesterol binding are consistent with proteins typical of multivesicular bodies and early endosomes [92]. Finally, the detection of regulators of cytoskeletal remodeling and trafficking, such as PAK1, underscores the capacity of EVsLysed to actively engage in signaling networks [2].

## 3 Conclusion

In the quest for effective EVs engineering strategies, the osmotic lysis method proposed in this study offers a promising, sustainable, and straightforward approach. By inducing membrane rupture through dilution in hypo‐osmotic conditions and allowing spontaneous reassembly of membrane fragments, this method not only increases particle yield but also redefines the physicochemical and biological identity of the resulting vesicle population. The lysed EVs emerge as a distinct nanomaterial platform, with favorable attributes for pharmaceutical applications, especially in drug delivery. To evaluate the engineering potential of this method, the lysed vesicle population was compared with naïve EVs and control vesicles.

A critical step in the proposed process is the selective dilution of EVs in bidistilled water, inducing a strong osmotic imbalance between the vesicle lumen, rich in cytosolic solutes, and the surrounding hypotonic environment. This leads to vesicle swelling, disruption of the phospholipid bilayers, and subsequent spontaneous reassembly into secondary vesicles. The self‐assembly of phospholipid bilayers into vesicular structures is a well‐established thermodynamically favorable process, driven by the minimization of interfacial free energy [66, 67]. Upon rupture, lipid membranes naturally reconfigure into closed bilayer structures, forming new vesicles to reduce the energetic cost of exposing hydrophobic domains to aqueous environments [64, 65, 103].

Such reassembly is consistent with the well‐characterized behavior of phospholipid bilayers, which tend to self‐organize into energetically favorable vesicular formations in aqueous environments to shield hydrophobic domains from water [67, 104]. The mechanism behind this transformation likely involves the influx of water into EVs upon osmotic imbalance between their salt‐containing luminal content and the surrounding bidistilled water. This rapid volume expansion can compromise the integrity of phospholipid bilayers, particularly in larger and more rigid vesicles. Fragmented membranes, due to their amphiphilic nature, thermodynamically favor reassembly into new vesicles by minimizing interfacial energy. The tension created at the membrane edges further promotes this fusion, enabling the formation of new vesicular structures.

Engineering EVs for therapeutic and diagnostic purposes often requires delicate interventions that preserve membrane integrity while enabling precise control over content and function. The osmotic lysis approach presented in this study provides a robust, scalable, and non‐disruptive strategy to remodel EVs at both structural and compositional levels. By exploiting the intrinsic self‐assembly properties of phospholipid membranes, this method enables the generation of a new class of vesicles (EVsLysed) with tailored features, enhanced functionality, and high translational potential.

Our findings demonstrate that osmotic shock enables selective depletion of the vesicular lumen, effectively removing endogenous proteins, nucleic acids, and other potentially immunogenic or tumorigenic components. Simultaneously, the membranes of the original vesicles reorganize into smaller, stiffer, and more numerous vesicles that maintain their native lipid and protein architecture. This rearrangement occurs without the need for chemical surfactants, sonication, electroporation, or other destabilizing treatments, ensuring high reproducibility and compatibility with in vivo applications.

EVsLysed combines several key advantages for pharmaceutical development. First, their increased abundance allows efficient scaling from limited biological sources, a crucial factor in clinical‐grade production, while maintaining a predominantly small‐EV size profile with well‐characterized and limited secondary subpopulations. Second, our data indicate an improvement in intrinsic purity, consistent with the elimination of luminal and co‐isolated contaminants, thereby addressing a common limitation in EV‐based formulations. Third, the engineered membranes are more structurally ordered and mechanically stable, properties that may support improved circulation time and cargo protection in physiological environments. Importantly, the clinical translation of EV‐based therapeutics is challenged by their immunological activity, as EVs have been reported to activate B cells and contribute to graft rejection, as well as to act as mediators of innate immune responses [16, 105, 106]. These findings indicate that patient‐to‐patient application of naïve EVs could carry intrinsic risks. In this context, EVs engineered through osmotic lysis, largely depleted of parental cargo, may represent a step toward safer and more versatile nanoplatforms, although their immunogenicity and rejection risk will need to be directly assessed in dedicated preclinical studies. Furthermore, the lysis process not only purges the vesicles of native cargo but also creates an opportunity for rational reloading. The ability to reconstitute vesicles with exogenous drugs, small molecules, or nanoparticles expands their applicability as customizable nanocarriers at a proof‐of‐concept level. In this regard, the vesicles generated via osmotic lysis may serve as both delivery vectors and relatively low‐background scaffolds, whose actual immunological profile and capacity to minimize off‐target effects will have to be established in future in vivo investigations. Another key benefit is the acquisition of enhanced targeting capacity. Although the preferential uptake of remodeled EVs by tumor cells is supported by correlative changes in membrane composition, including phosphatidylserine exposure and enrichment of membrane‐associated proteins, the specific receptors and internalization pathways responsible for this behavior remain to be determined and will need to be addressed in future receptor‐blocking and pathway‐focused studies. This passive tropism, coupled with the potential for further surface engineering, positions EVsLysed as a powerful platform for precision drug delivery. Moreover, the selective recovery of luminal content following lysis opens new avenues for diagnostic applications, enabling parallel analysis of disease biomarkers.

Technically, the approach is remarkably accessible. It requires only standard laboratory instrumentation, such as centrifuges and basic filtration units, and can be adapted for sterile workflows. Its simplicity and versatility make it amenable to a wide range of cell sources, including tumor‐derived EVs whose oncogenic risks are mitigated through cargo depletion. The same core protocol can thus be applied to design vesicles tailored to distinct diseases, payloads, and therapeutic objectives.

In summary, osmotic lysis provides a promising and experimentally validated approach for engineering extracellular vesicles in a B lymphocyte–derived model, enabling selective cargo removal and membrane remodeling while preserving vesicle integrity under the conditions tested. In this work, we specifically focused on extracellular vesicles derived from immortalized B lymphocytes, chosen as a representative model of circulating immune cells and as a source whose baseline physiology and tropism are well characterized [107]. Within this framework, the osmotic remodeling protocol should be regarded as a proof‐of‐concept platform, whose underlying physical principle, namely the generation of a controlled osmotic gradient across the vesicle membrane, is in principle adaptable to extracellular vesicles from other cellular sources and to distinct disease settings, provided that the treatment conditions are appropriately optimized for each context. Within the limits of the present in vitro data, osmotically remodeled vesicles can therefore be regarded as a candidate nanovesicle platform with features that may be relevant for future applications such as drug delivery, nanodiagnostics, and immune modulation, which will, however, require dedicated preclinical validation in disease‐specific models. On this basis, future studies will need to extend this strategy to additional extracellular vesicle origins and therapeutic targets, and to systematically assess in vivo performance, pharmacokinetics, biodistribution, immunogenicity, efficacy, and safety in clinically relevant models, in order to determine the actual suitability of these engineered vesicles for translation into clinical‐grade therapeutics and diagnostics.

From a translational standpoint, the workflow described here is intrinsically amenable to scale‐up and implementation under clinically compliant conditions, as it relies exclusively on aqueous media, osmotic gradients, and standard unit operations such as centrifugation, buffer exchange, and filtration. The same sequence of steps can, in principle, be transferred to closed and sterile platforms based on tangential‐flow filtration or dialysis‐like buffer exchange, which are already established in current good manufacturing practice (cGMP) bioprocessing. While a full manufacturing and regulatory assessment lies beyond the scope of this study, these features suggest that osmotic remodeling could be integrated into existing EV production pipelines as a relatively simple conditioning module, provided that future work systematically addresses process control, batch‐to‐batch consistency, and critical quality attributes under clinically relevant scale and compliance constraints.

## 4 Experimental Section/Methods

All isolation, processing, and characterization steps were performed in full accordance with the MISEV2023 guidelines issued by the International Society for Extracellular Vesicles (ISEV) [108]. Key experimental controls and reporting requirements were included. We have submitted all relevant data of our experiments to the EV‐TRACK knowledgebase (EV‐TRACK ID: EV250086) [109].

### 4.1 Cell Cultures

B‐lymphocytes were purchased from the cell bank of the IRCCS Ospedale Policlinico San Martino of Genova (Italy). Cells were cultured in RPMI 1640 cell culture medium (Euroclone, Pero (MI), Italy) supplemented with 20% of fetal bovine serum (FBS, Euroclone) inactivated at 56°C for 30 min, 1% L‐glutamine 200 mM (Q, Euroclone), 1% of MEM Non‐Essential Amino Acids solution (MEM, 100X, ThermoFisher Scientific, Waltham, MA, USA) and 1% of penicillin/streptomycin (P/S, 10000 units penicillin and 10 mg streptomycin/mL, Euroclone) maintaining the cell density between 9 × 10^4^ and 9 × 10^5^ cells/mL.

Raji cell line was purchased from ATCC (ATCC, Manassas, VA, USA) and cells were cultured in RPMI 1640 culture medium, supplemented with 10% of FBS, 1% of Q 200 mM, 2% of P/S, and the cell culture density was maintained between 2 × 10^5^ and 9 × 10^5^ cells/mL.

### 4.2 Extracellular Vesicles Isolation

To isolate EVs from B‐lymphocytes, 1.5 × 10^5^ cells/mL were cultured for 72 h in RPMI 1640 cell culture medium with 20% of depleted FBS, 1% Q, 1% MEM, and 1% P/S. To obtain depleted FBS, heat‐inactivated FBS was ultracentrifuged overnight at 100000 g at 4°C using an Optima MAX‐XP ultracentrifuge and an MLA‐50 rotor (Beckman Coulter, Brea, CA, USA). EVs were isolated according to a modified sterile differential ultracentrifugation protocol [107, 110]. Specifically, after 72 h culture, lymphocytes conditioned culture media were collected and centrifuged at 150 g for 10 min to discard viable cells, then the obtained supernatant centrifuged at 2000 g for 20 min to eliminate dead cells, and the supernatant centrifuged at 10000 g for 30 min to discard debris. The obtained supernatant was transferred to ultracentrifuge polypropylene tubes and ultracentrifuged at 100 000 g for 70 min. The obtained pellet was recovered and washed in cold 0.1 µm filtered phosphate‐buffered saline (1X, PBS, ThermoFisher Scientific) solution at 100 000 g for 60 min. All the centrifuge and ultracentrifuge steps were carried out at 4°C. The final pellet was recovered by resuspension in 0.1 µm filtered saline solution (0.9% NaCl, Eurospital, Trieste, Italy). After isolation, EVs aliquots were stored at −80°C for further use.

### 4.3 Extracellular Vesicles Osmotic Lysis

EVs were osmotically lysed using the process described in the patent [111]. Specifically, EVsLysed were produced by diluting 1:50 (v/v) nEVs in bidistilled water, then the obtained EVs solution was vortexed for 3 min, incubated at 37°C for 30 min on a thermostatic orbital shaker at 500 rpm, vortexed again for 1 min, and concentrated via ultrafiltration with a 50 kDa Amicon Ultra centrifugal filter.

In this experimental setup, EVsCtrl resuspended and diluted 1:50 in saline solution (0.9% NaCl) served as the negative control for the EVsLysed condition, allowing experimental direct comparison of the lytic effect independently of dilution or processing steps.

### 4.4 EVs Characterization Methods

The different characterization methods used for the analysis of nEVs, EVsCtrl, and EVsLysed were carried out according to the minimal requirements recommended by the International Society for Extracellular Vesicles (ISEV) [112].

### 4.5 Nanoparticle Tracking Analysis

Concentration and size distribution of nEVs, EVsLysed, and EVsCtrl were assessed using the NTA technique through a Nanosight NS300 (Malvern Panalytical, Malvern, UK) with a laser beam (λ = 505 nm) and an automated syringe pump. To provide reliable measurements, samples were diluted in suitable dilution media (saline or bidistilled water, up to 500 µL), maintaining the particles/frame index between 20 and 100. For the analyzes, three videos of 60 s each were acquired, with an infusion pump rate of 50 A.U., screen gain at 1, and the camera level between 15 and 16. The videos were then analyzed by the NTA 3.4 software with the detection threshold set at 5. Different experiments were carried out at least eight times with three technical replicates each and analyzed with the *t*‐test.

### 4.6 Total Protein Content Assay

Total protein concentration of each EVs sample was measured at least six times and three technical replicates by Bradford assay (Bio‐Rad Protein Assay Dye Reagent Concentrate, Bio‐Rad, Hercules, CA, USA) according to the manufacturer's instructions. Bradford assay was performed in a clear 96‐well plate, and 595 nm absorbance was measured using a Multiscan Sky spectrophotometer (ThermoFisher Scientific).

### 4.7 Western Blot

For the western blot analysis, 5× Laemmli buffer was added to the EV samples, which were then boiled for 5 min at 95°C. Proteins were separated via SDS‐PAGE using a 4%–20% Mini‐PROTEAN TGX Precast Protein Gel (Bio‐Rad) and transferred onto a nitrocellulose membrane (Bio‐Rad, Trans‐Blot Turbo). The membrane was blocked in Tris‐buffered saline (TBS)‐ TRIS T containing 1% BSA for 1 h and incubated overnight at 4°C with the primary antibodies: anti‐CD81 and anti‐CD63 (1:1000, Santa Cruz Biotechnology, Dallas, TX, USA) and anti‐TSG101 (1:1000, Novus Biologicals, Toronto, Canada). After three washes with TBS‐T, the membranes were incubated with horseradish peroxidase‐conjugated secondary antibodies (1:3000 in TBS‐T with 1% BSA) for 1 h. Signals were developed using the Bio‐Rad Clarity Western ECL Substrate and visualized with the Chemidoc XRS+ system (Bio‐Rad). For Dot Blot analysis, EVs diluted 1:1 and 1:2 in PBS buffer (1 µL) were spotted on a nitrocellulose membrane and allowed to dry at RT for 1 h. Membranes were then blocked in TBS containing 1% BSA in the absence of 0.1% (v/v) Tween‐20 for 1 h at RT and incubated overnight at 4°C with the primary antibody anti‐TSG101 (1:1000, Novus Biologicals, Toronto, Canada) diluted 1:1000 in the same buffer. After three washes with TBS, membranes were incubated with horseradish‐peroxidase ‐conjugated proper secondary antibodies diluted in TBS 1% BSA for 1 h at RT. Blots were detected as described above.

### 4.8 Single Molecule Array

For the SiMoA analysis, the conjugation of beads to antibodies was performed following the Quanterix Homebrew kit instructions, utilizing the recommended buffers (Quanterix, Billerica, MA, USA). Carboxylate paramagnetic beads (150 µL, 2.8 × 10^9^ particles/mL) were washed three times with Bead Wash Buffer (300 µL, Quanterix, PBS with detergent). After each wash, the beads were centrifuged and placed on a magnetic separator for 1 min to aspirate the supernatant. The beads were then washed an additional three times with Bead Conjugation Buffer (300 µL, Quanterix, 50 mM MES buffer, pH 6.2) and subsequently activated with 1‐etil‐3‐(3‐dimetilaminopropil)carbodiimide (0.3 mg/mL, EDC) for 30 min at 4°C under continuous mixing/shaking. The antibody (80 µg, CD63 and CD81) was buffer‐exchanged using a 50 kDa Amicon filter and recovered in Quanterix Bead Conjugation Buffer. After buffer exchange, antibody solution (300 µL, 0.2 mg/mL) was added to the activated paramagnetic beads and incubated for 2 h at 4°C with continuous mixing/shaking. Following the conjugation step, the beads were washed twice with Bead Wash Buffer and subsequently blocked with Bead Block Buffer (Quanterix, PBS with BSA) for 45 min at room temperature with continuous mixing/shaking. After blocking, the beads were washed three times with Bead Diluent and stored at 4°C until use.

A pan‐tetraspanin bead solution was prepared at a concentration of 2 × 10^7^ beads/mL in Bead Diluent. Detector antibody solutions (biotinylated CD63 and CD81 antibodies from Ancell) were prepared at a concentration of 0.3 µg/mL in Homebrew Sample Diluent (Quanterix). Similarly, EV samples were diluted 1:10 in Homebrew Sample Diluent. Beads (25 µL) were transferred into a 96‐well microwell plate, mixed with diluted sample (100 µL), and incubated for 30 min at 25°C with agitation at 800 rpm [113]. After incubation, the beads were washed using an automated plate washer and then incubated for 10 min with the detector antibody solution (100 µL). Following another wash, the beads were incubated for 10 min with Streptavidin‐β‐D‐Galactosidase (SBG) solution (150 pM, prepared in SBG Diluent, Quanterix). After the SBG incubation step, the plate was washed again and inserted into the Quanterix SR‐X instrument for analysis, during which resorufin b‐galactopyranoside (RGP) was automatically added. Data were analyzed and processed using Reader Software Simoa 1.1.0.

### 4.9 Electron Microscopy

The morphology of EVs was investigated with transmission and field emission scanning electron microscopy.

To visualize EVs samples under the TEM, carbon and formvar‐coated 400 mesh grids were glow‐discharged with an Edward E12E vacuum coating unit immediately before use. A drop of sample (7 µL) was deposited onto the grid, let 5 min for adsorption onto the grid surface, rinsed several times with water, and negatively stained with aqueous 0.5% w/v uranyl acetate. The excess solution was removed using filter paper.

TEM observation was carried out using a FEI Tecnai BioTwin G2 Spirit microscope. The specimens were observed using an acceleration voltage of 80 kV and an emission current of approximately 25 micro‐Ampere in the bright field; data were collected with a CCD camera.

For SEM EVs samples investigations, a single drop of the different EVs samples was deposited on a silicon wafer and let dry at room temperature; then the wafers were mounted on aluminium stubs and coated with a thin layer (∼5 nm) of platinum using a sputter coater Quorum Q150T S. Images were acquired with a field emission scanning electron microscope (Supra 40, Zeiss, Oberkochen, Germany) at 5 kV.

### 4.10 Atomic Force Microscopy

AFM micrographs were recorded following a procedure described elsewhere [75]. Images were acquired in PeakForce mode on a Multimode 8 microscope (Bruker, Billerica, MA, USA) equipped with Scanasyst Fluid+ probes (Bruker), a Nanoscope V controller, a type JV piezoelectric scanner, and a sealed fluid cell. Briefly, each EV sample (5 µL) was deposited on poly‐L‐lysine (PLL) functionalized glass coverslips and left to adsorb for 60 min at 4°C, then inserted in the fluid cell without further rinsing. Quantitative morphometry was performed via Gwyddion 2.61 [114] and custom Python scripts. Two parameters were calculated for each individual object deposited on the surface: the equivalent spherical diameter and the contact angle it assumed once adsorbed to the surface. The latter was demonstrated to be directly proportional to the mechanical stiffness of intact vesicles [74]. The total deposited volume of material per square micrometer was used to estimate the relative number of particles in different samples.

### 4.11 Lipidomic Analysis

Lipid extraction from EVs was performed using a modified Matyash method with two cold organic solvents [115]. Vesicles were lysed by sonication, followed by the addition of a methanol mixture containing deuterated standards (5 µL, Splash Lipidomix, Merck, Darmstadt, Germany). Then, cold methanol (225 µL, VWR, Milano, Italy) was added, and the samples were vortexed for 10 s. Afterward, cold methyl tert‐butyl ether (750 µL) was added, and the solution was vortexed again for 10 s. The samples were incubated at 4°C for 6 min under agitation at 2000 rpm in a thermomixer. To induce phase separation, water (100 µL, LC‐MS water, VWR, Milano, Italy) was added, and samples were vortexed for 10 s. The mixture was centrifuged at 14000 rpm for 2 min at 4°C, and 500 µL of the upper organic phase was carefully collected and evaporated using a SpeedVac. Finally, the dried lipid extract was reconstituted in methanol (50 µL) containing the internal standard CUDA at a final concentration of 12.5 ng/mL.

For the analysis of the reconstituted lipids, a UHPLC Vanquish system (ThermoFisher Scientific) coupled with an Orbitrap Q‐Exactive Plus mass spectrometer (ThermoFisher Scientific) was employed. Lipid separation was performed using a reverse‐phase column (ACQUITY UPLC CSH C18 1.7 µm 2.1×150 mm) maintained at 45°C with a flow rate of 0.260 mL/min.

In the ESI positive mode, mobile phase A consisted of acetonitrile/water (60:40, v/v) with 10 mmol ammonium formate, while mobile phase B was isopropanol/acetonitrile (90:10, v/v), modified with 10 mmol ammonium formate and 0.1% formic acid. In the ESI negative mode, the same solvents were used, but ammonium acetate (10 mmol) replaced ammonium formate. The gradient program was as follows: 0–2 min, 30% to 43% B; 2–2.1 min, 43% to 55% B; 2.1–12 min, 55% to 65% B; 12–18 min, 65% to 85% B; and 18–20 min, 85% to 100% B. The column was maintained at 100% B for 5 min before re‐equilibration at 30% B for an additional 5 min, with a total runtime of 30 min.

Mass spectrometry analysis was conducted in both positive and negative ion modes with a source voltage of 3.5 kV for positive mode and 2.8 kV for negative mode. The capillary temperature was set to 320°C, while sheath gas and auxiliary gas flows were maintained at 40 arb and 3 arb, respectively, and the S‐lens RF level was set to 50. Data were acquired in a data‐dependent (ddMS2) top‐10 scan mode, with survey full‐scan MS spectra (m/z 80–1200) at a resolution of 70 000 and an AGC target of 1 × 10^6^. MS/MS fragmentation used high‐energy c‐trap dissociation (HCD) with a resolution of 17 500, AGC target of 1 × 10^5^, and stepped normalized collision energies (NCE) of 15, 30, and 45.

The injection volume was 3 µL. For accurate mass‐based analysis, regular Lockmass and inter‐run calibrations were used. An exclusion list for background ions was generated by testing the same procedural sample for both positive and negative ESI modes. Quality control was ensured by analyzing pooled samples before, at the beginning, and at the end of the batches by using blanks to check for residual interference from internal standards, which include a series of analyte classes (Avanti SPLASH Lipidomix) and an internal standard (CUDA) prior to liquid chromatography‐mass spectrometry (LC–MS) analysis. Raw data acquired from lipidomic untargeted analysis were processed with MSDIAL software (Yokohama City, Kanagawa, Japan), version 4.24. Peaks were detected, MS2 data were deconvoluted, compounds were identified, and peaks were aligned across all samples. For quantification, the peak areas for the different molecular species detected were normalized using the deuterated internal standard for each lipid class. To obtain an estimated concentration expressed in nmol/mL, the normalized areas were multiplied by the concentration of the internal standard. An in‐house library of standards was also used for lipid identification. Statistical analyzes were performed with MetaboAnalyst 6.0 (www.metaboanalyst.org). The analyzes were carried out on two independent biological replicates with three technical replicates each.

### 4.12 Proteomic Analysis

For proteomic analysis, vesicles were lysed with one freeze‐thaw cycle, with RIPA buffer and ultrasonication. Proteins were then precipitated with cold acetone and resuspended. Proteins were then reduced in NH_4_HCO_3_ (25 µL, 100 mM) with DTT (2.5 µL, 200 mM, Merck) at 60°C for 45 min and next alkylated with iodoacetamide (10 µL, 200 mM, Merck) for 1 h at RT in dark conditions. Iodoacetamide excess was removed by the addition of DTT (200 mM). Proteins were then digested with trypsin. The digests were dried by Speed Vacuum and then desalted. Digested peptides were analyzed on an Ultimate 3000 RSLC nano coupled directly to an Orbitrap Exploris 480 with a High‐Field Asymmetric Waveform Ion Mobility Spectrometry System (FAIMS) (all from Thermo Fisher Scientific). Samples were injected onto a reversed‐phase C18 column (15 cm × 75 µm i.d., Thermo Fisher Scientific) and eluted with a gradient of 6% to 95% mobile phase B over 41 min by applying a flow rate of 500 nL/min, followed by an equilibration with 6% mobile phase B for 1 min. The acquisition time of one sample was 41 min, and the total recording of the MS spectra was carried out in positive resolution with a high voltage of 2500 V and the FAIMS interface in standard resolution, with a CV of ‐45 V. The acquisition was performed in data‐independent mode (DIA): precursor mass range was set between 400 and 900, isolation window of 8 m/z, window overlap of 1 m/z, HCD collision energy of 27%, orbitrap resolution of 30000, and RF Lens at 50%. The normalized AGC target was set to 1000, the maximum injection time was 25 ms, and the microscan was 1. For DIA data processing, DIA‐NN (version 1.8.1) was used: the identification was performed with “library‐free search” and “deep learning‐based spectra, RTs and IMs prediction” enabled. Enzyme was set to Trypsin/P, precursors of charge state 1–4, peptide lengths 7–30, and precursor m/z 400–900 were considered with a maximum of two missed cleavages. Carbamidomethylation on C was set as a fixed modification, and Oxidation on M was set as a variable modification, using a maximum of two variable modifications per peptide. FDR was set to 1%.

The mass spectrometry proteomics data have been deposited to the ProteomeXchange Consortium via the PRIDE partner repository with the dataset identifier PXD062028.

Statistical analyses were performed with MetaboAnalyst 6.0 (www.metaboanalyst.org). Statistical Analysis [single factor] function was used to build volcano plots, principal component analysis (PCA), partial least squares‐discriminant analysis (PLS‐DA), and heatmaps. Bioinformatic analysis was carried out using DAVID software (https://david.ncifcrf.gov/tools.jsp) and Ingenuity Pathway Analysis (Qiagen, Hilden, Germany). The analyses were carried out on three independent biological replicates with three technical replicates each.

### 4.13 Extracellular Vesicles Uptake Characterization

The uptake of EVsLysed and EVsCtrl was evaluated at different concentrations (5 and 10 µg/mL) and time points (2, 4, and 24 h). The nEVs sample was labelled adding Wheat Germ Agglutinin, Alexa Fluor 488 Conjugate (1 µL, WGA488, λ_ex_ = 650 nm, 1 mg/mL in PBS, ThermoFisher Scientific) to EVs (100 µL) and the solution was incubated for 30 min at 37°C at 500 rpm in dark. Then, labelled nEVs were treated as previously described in section 2.3 for the preparation of both lysed and control vesicles. After the ultrafiltration in 50 kDa Amicon filters, a step suitable to remove the unbound dye and concentrate the EVs, the two eluted solutions, EVsLysed and EVsCtrl, were resuspended in the specific cell culture medium for each line to the final concentration of 5 and 10 µg/mL.

To evaluate the internalization of EVsLysed and EVsCtrl in B lymphocytes and Raji cell lines, 2 × 10^5^ cells for each mL of treatment were centrifuged, pellets resuspended in the treatment solutions, and plated in a non‐treated 96‐well plate (250 µL for each well). After 2, 4, and 24 h of incubation, cells were washed in sterile 0.22 µL filtered PBS (1 mL, 1X) and centrifuged at 150 g for 10 min at RT.

Then, the upper phase was discarded, and the cell pellet was gently suspended in PBS (300 µL, 1X), transferred to FACS tubes, and immediately acquired on a BD Accuri C6 Flow Cytometer (BD Biosciences, Franklin Lakes, NJ, USA). Cells were gated on physical parameters (forward and side scatter) and analyzed for Alexa‐488 associated fluorescence (FL‐1 channel). A minimum of 5000 events (cells) were collected for each sample, and the mean fluorescence intensity was analyzed relative to control samples by BD Accuri C6 software. The experiment was carried out three times, and, when possible, three technical replicates were acquired for each sample.

### 4.14 Characterization of the Release of the Native Nucleic Acid Content

To verify the effectiveness of the lysis method in emptying the naïve vesicles from their inner cargo, the presence of nucleic acids inside the supernatant after the lysis was detected by exploiting their absorbance length.

To this aim, EVs (starting from 10 µg in protein content) were subjected to the lysis protocol. After the incubation at 37°C, bidistilled water and saline were added to EVsLysed and EVsCtrl, respectively, to reach the volume to fill the ultracentrifuge tube. The two samples were ultracentrifuged at 100 000 g for 60 min, the supernatants of EVsLysed and EVsCtrl were collected and concentrated using a BUCHI‐re111 rotary evaporator (Buchi, Flawil, Switzerland), with a water bath type B 461 at 37°C. After the evaporation, the residual pellet was resuspended in bidistilled water (1 mL), and the nucleic acid content of EVsLysed and EVsCtrl was measured using a Synergy HTX Multi‐Mode Microplate Reader with the Take3 microvolume plate (BioTek, Agilent Technologies, Santa Clara, CA, USA) at 260 and 280 nm. The experiment was repeated twice with at least four technical replicates.

### 4.15 Exogenous Gold Nanoparticle Engineering and Characterization

Based on the observed differences in uptake behavior of the various EV populations, we investigated their interaction with exogenous AuNPs, aiming to evaluate whether their remodeling affected nanoparticle loading efficiency. To test the different affinity between the EVsLysed and EVsCtrl, 5 nm gold nanoparticles (AuNPs, 0.01% Au, NANOCS, NEW YORK, NY, USA) were used for the TEM imaging, and 5 nm Cy5‐labelled AuNPs (Cy5AuNPs, excitation/emission 650/670 nm, 0.5 mg/mL, NANOCS) were used for the flow cytometric analysis. In detail, during the preparation of EVsLysed and EVsCtrl, AuNPs and Cy5AuNPs were added to the hypo‐osmotic (bidistilled water) and to the isosmotic (saline physiological) solutions in an EVs:gold nanoparticles ratio of 1:5000 to favor the interaction between them. This ratio was chosen as an indicative approximation, based on the theoretical volumetric capacity of a 100 nm EV to accommodate up to ∼5000 AuNPs of 2.5–5 nm. While this does not imply actual luminal encapsulation of that number of particles, it provides a conceptual framework to explore how remodeling may influence nanoparticle loading and localization (membrane‐bound vs intraluminal).

This ratio was calculated using Equation (1):

(1) $$n^{\circ }\mathrm{AuNPs}=\frac{\frac{4}{3}\pi R_{\mathrm{EV}}^{3}}{\frac{4}{3}\pi r_{\mathrm{AuNP}}^{3}}\;*\;\mathrm{pf}$$

Considering that R_EV​_ is the radius of the EV (50 nm), r_AuNP_​ is the radius of a single AuNP (2.5 nm), pf is the packing factor, accounting for the spatial inefficiency in the arrangement of spherical particles (typically ∼0.64 for random close packing).

The interaction between both EVsLysed and EVsCtrl and the AuNPs and Cy5AuNPs was tested, respectively, by TEM and flow cytometry.

For the TEM analysis, the samples were prepared as reported above.

For the flow cytometric analysis, after the interaction with Cy5AuNPs, EVsLysed and EVsCtrl (2.5 µg) were coupled for 15 min at RT with Aldehyde/Sulfate Latex Beads (5 µL, 4% w/v, 3 µm, Thermo Fisher). Then, PBS was added to a final volume of 500 µL, and the coupling continued for 2 h at RT on a tube rotator with a fixed speed of 20 min^−1^. Samples were washed three times for 3 min at 4000 rpm and resuspended in PBS (250 µL). For the cytofluorimetric analysis, 5 µL were diluted in PBS (200 µL) and analyzed. Control beads were used to adjust instrument voltages and gate bead population to exclude debris and impurity derived from the buffer solution. The Cy5‐associated fluorescence was detected on 5000 gated events, and results were analyzed in terms of median fluorescence intensity. The experiment was repeated ten times.

### 4.16 Statistical Analysis

Plotted data are the mean ± standard error (S.E.) or the mean ± standard deviation (S.D.). Unless otherwise specified, the statistical analysis between the treatment groups was performed using the ANOVA or the t‐test tools in the SIGMA Plot software's data analysis package. * p < 0.05 and ** p ≤ 0.001 were considered significant.

## Funding

This research did not receive any specific grant from funding agencies in the public, commercial, or not‐for‐profit sectors.

## Conflicts of Interest

The authors T.L., F.S., and R.P. are co‐inventors on patent number: IT102021000027725 ‐ PCT/IB2022/060343 that covers aspects of the methods described in this article.

## Data Availability

All isolation, processing, and characterization steps were performed in full accordance with the MISEV2023 guidelines issued by the International Society for Extracellular Vesicles (ISEV). Key experimental controls and reporting requirements were included. You may access the submission of experimental parameters to the EV‐TRACK knowledgebase via the following URL: http://evtrack.org/review.php, using the ID EV250086 and the last name of the first author (Susa). The mass spectrometry proteomics data have been deposited to the ProteomeXchange Consortium via the PRIDE partner repository with the dataset identifier PXD062028.
